# Triplet states enable efficient photocatalytic hydrogen evolution in star-shaped truxene-based nanoparticles

**DOI:** 10.1039/d5sc09380e

**Published:** 2026-02-05

**Authors:** Andjela Brnovic, Gaurav Kumar, Martin Axelsson, Bin Cai, Mariia V. Pavliuk, Lars Kloo, Leif Hammarström, Haining Tian

**Affiliations:** a Department of Chemistry, Ångström Laboratory, Uppsala University Uppsala SE 75120 Sweden haining.tian@kemi.uu.se; b Applied Physical Chemistry, Department of Chemistry, KTH Royal Institute of Technology SE-10044 Stockholm Sweden

## Abstract

We have developed two new star-shaped donor–acceptor oligomers, named TxBT and TxNT, with a truxene donor core and either 2,1,3-benzothiadiazole (BT) or a naphtho[1,2-*c*:5,6-*c*′]bis[1,2,5]thiadiazole (NT) unit, respectively. Femtosecond transient absorption spectroscopy suggested that both oligomer nanoparticles (NPs) generate long-lived triplet charge-transfer (CT) states following photoexcitation, which undergo reductive quenching by ascorbate. TxNT NPs generate a larger population of reduced species that accumulate and escape recombination compared to TxBT NPs, indicating more efficient charge separation. TxNT NPs show significantly higher hydrogen evolution rate (54 mmol h^−1^ g^−1^) compared to TxBT NPs, which is comparable to the performance of the most efficient heterojunction polymer NP systems. Additionally, morphological analysis revealed that Pt deposition was significantly lower on TxBT than on TxNT NPs. These findings highlight the critical role of triplet CT states, tuning molecular energy levels, optimizing excited-state dynamics, and engineering NP architecture to increase photocatalytic hydrogen evolution of organic photocatalysts. To our knowledge, this is the first report where triplet CT states can mediate photocatalytic hydrogen evolution in donor–acceptor oligomer NPs.

## Abstract 2

Two new star-shaped donor–acceptor oligomers, named TxBT and TxNT, with a truxene donor core and either 2,1,3-benzothiadiazole (BT) or a naphtho[1,2-*c*:5,6-*c*′]bis[1,2,5]thiadiazole (NT) unit, respectively, have been developed for photocatalytic hydrogen production.

## Graphical abstract

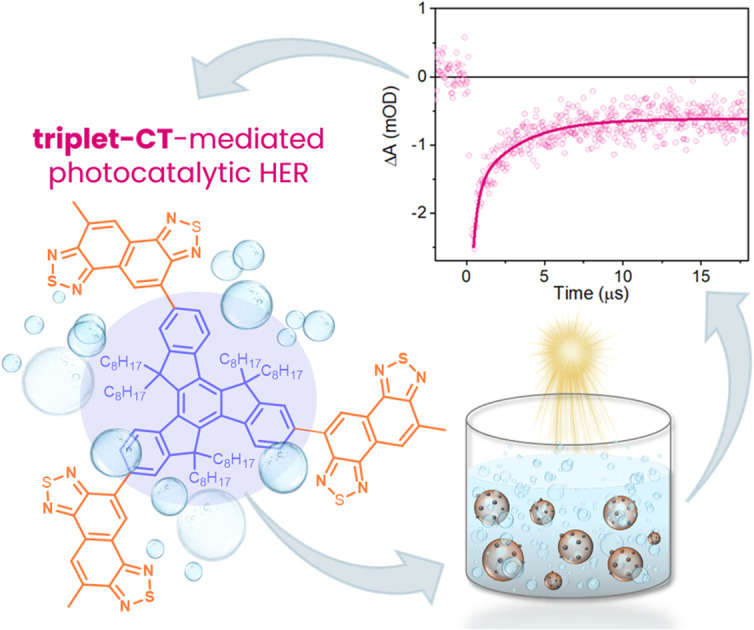

## Introduction

Among artificial photosynthetic approaches for solar-to-chemical energy conversion, photocatalysis has attracted considerable attention due to its simplicity, scalability, and cost-effectiveness.^1^ Organic semiconductors, such as π-conjugated polymers, are promising photocatalysts due to their tunable optical properties, absorption extending into the visible region, high extinction coefficients, and composition of only earth-abundant elements.^2,3^ However, one of the requirements for achieving high photocatalytic activity is efficient charge separation which is often limited by large exciton binding energies in organic materials.^4,5^

To address this limitation, conjugated polymers can be self-assembled into nanoparticles (NPs) which, unlike bulk polymers, can be dispersed in water. Additionally, confining polymers within NPs shortens exciton migration distances compared to bulk polymers, increasing the probability that excitons will reach the NP–water interface within their lifetime. When excitons reach the NP–water interface, the high dielectric constant of water promotes charge separation by screening Coulomb interactions between tightly bound charges.^6–10^

Linearly conjugated polymers have been extensively studied for the photocatalytic hydrogen evolution reaction (HER), with performance enhancements achieved through increasing the hydrophilicity of polymer backbones and employing donor–acceptor (D–A) architectures to reduce exciton binding energies and facilitate intramolecular charge transfer.^11–18^ More recently, embedding these polymers in NPs, either as single component or D–A heterojunctions, has shown that nanoscale morphology strongly influences HER activity.^10,19,20^ In particular, NPs with porous shells and proton-conducting channels have demonstrated improved HER performance.^20^

**Fig. 1:**
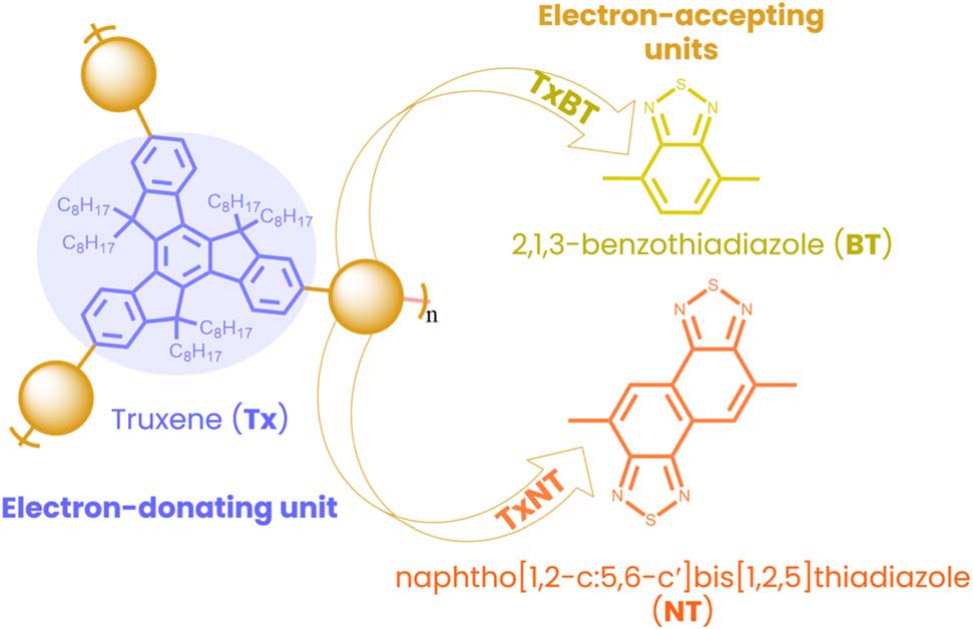
Molecular structures of star-shaped D–A oligomers TxBT and TxNT with an electron-rich Tx core connected through conjugated arms to electron-poor acceptor units. TxBT contains terminal electron-accepting BT units, while TxNT includes NT units.

Here, we have designed and synthesized two new star-shaped D–A oligomers, named TxNT and TxBT, each consisting of an electron-rich truxene (Tx) core and electron-poor units at the ends of their conjugated arms. TxNT incorporates naphtho[1,2-*c*:5,6-*c*′]bis[1,2,5]thiadiazole (NT) as electron-accepting units, while TxBT has 2,1,3-benzothiadiazole (BT) units (Fig. 1). Structurally related Tx-based materials have been reported previously in different contexts. For example, a TxBT-based Tx derivative has been described as part of a porous polymer network, targeting different applications other than the photocatalytic HER.^21^ Tx-based materials have also been explored in hybrid photocatalytic systems, such as Tx-TiO_2_ composites for the HER.^22^ In contrast, our work focuses on single-component, purely organic Tx-based NPs for the photocatalytic HER. While the Tx core is known for its facile functionalization and high chemical stability, the NT moiety has not previously been explored in photocatalysis and is expected to provide strong electron-withdrawing functionality, increase light absorption, enhance interchain packing, and improve charge carrier mobility.^23–25^ Compared to linear analogues, star-shaped D–A architectures offer strong absorption, improved charge mobility pathways, better photostability and enhanced intramolecular charge transfer. Star-shaped architectures have been studied for organic photovoltaics and bioimaging applications but remain largely unexplored for photocatalysis, especially for hydrogen evolution.^26,27^

These oligomers were prepared into water-dispersible TxNT and TxBT NPs using an amphiphilic polystyrene-poly(ethylene glycol) carboxylic acid surfactant (PS-PEG-COOH) by a nanoprecipitation method. We systematically compared their morphology, optical and electronic properties, as well as HER performance under visible light illumination. Transient absorption spectroscopy across femtosecond to microsecond timescales was used to investigate the excited-state dynamics and to identify the nature of long-lived intermediates. Our findings highlight a triplet-CT-mediated mechanism which is, to our knowledge, the first experimental support of a triplet CT state actively participating in the photocatalytic HER within oligomer-based NPs. These findings provide design principles for advancing organic photocatalysts for solar hydrogen production.

## Results and discussion

### Synthesis and characterization of TxBT and TxNT oligomers

Both oligomers were synthesized *via* Suzuki–Miyaura coupling (details in the SI). Successful polymerization was confirmed by proton and carbon-13 nuclear magnetic resonance (^1^H NMR and ^13^C NMR) spectroscopy while number-average molecular weights (*M*_n_) and molecular weight distribution PDI (*M*_w_/*M*_n_) of the polymers were determined by gel permeation chromatography (GPC) according to the procedure in the SI. *M*_n_ values were 9300 g mol^−1^ for TxNT and 8300 g mol^−1^ for TxBT, with corresponding PDI values of 1.8 and 1.6, respectively.

**Fig. 2:**
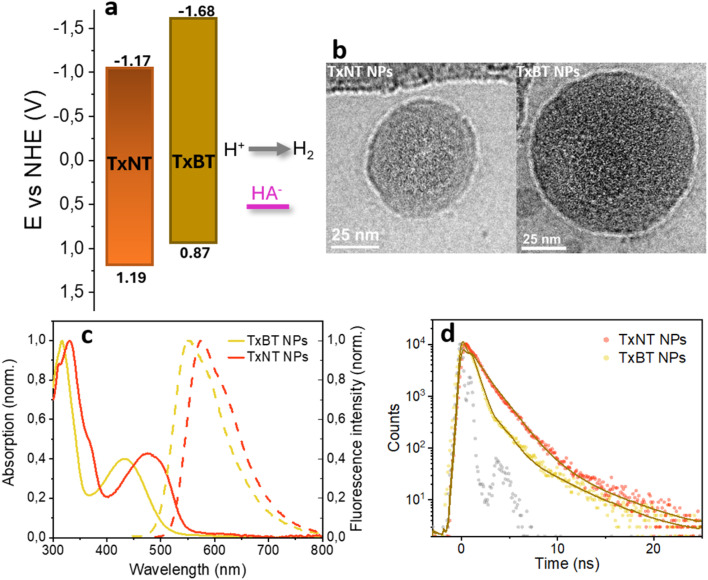
(a) Energy level diagram of TxNT and TxBT oligomers showing favourable alignment for proton reduction and regeneration by ascorbate at pH 4.2. (b) Cryo-TEM images of TxNT NPs and TxBT NPs. (c) Normalized UV-vis absorption (solid lines) and PL (dashed lines) spectra of TxNT and TxBT NPs. (d) TCSPC fluorescence decays of TxNT and TxBT NPs with corresponding exponential fits.

Cyclic voltammetry (CV) measurements in THF (Fig. S5) were used to determine the standard reduction potentials of the oligomers while the zero–zero transition energy (*E*_00_) was determined from the intersection of normalized absorption and fluorescence (PL) spectra in THF (2.36 and 2.55 eV, for TxNT and TxBT, respectively). The reduction potential of the excited state (E^*/−^) was estimated from the determined reduction potential of the ground state (E^0/−^) and *E*_00_. Fig. 2a shows favourable energy level alignment for proton reduction and regeneration by ascorbate at pH = 4.2. The stronger electron-accepting character of the NT units relative to the BT units results in a significantly lower E^0/−^ of TxNT compared to TxBT and narrower bandgap. E^*/−^ of TxNT is 0.32 V more positive than that of TxBT, therefore providing a larger driving force for reduction by ascorbate in the TxNT oligomer.

### Morphological properties

Given the favourable molecular structures and redox properties of the oligomers, we further investigated their potential as photocatalysts for hydrogen generation. To obtain water-dispersible photocatalysts, oligomer NPs with PS-PEG-COOH were prepared under ultrasonication (see the SI for details). The preparation procedure yielded NPs with similar hydrodynamic diameters, about 80 nm for both, TxNT and TxBT NPs, as determined by dynamic light scattering (DLS) (Fig. S6). Cryogenic transmission electron microscopy (cryo-TEM) showed that both TxNT and TxBT NPs are quasi-spherical and largely amorphous, with no evidence of highly crystalline internal features (Fig. 2b and S7).

### Optical properties: UV-vis absorption and photoluminescence

The UV-vis absorption and photoluminescence (PL) spectra of the investigated oligomers in NPs and in THF are shown in Fig. 2c and S8. Both oligomers in THF exhibited broad absorption up to 575 and 525 nm for TxNT and TxBT, respectively, due to intramolecular charge transfer (CT) from the D–A architecture. Despite aggregation into NPs, both TxNT and TxBT show minimal spectral shifts in their absorption and PL maxima when comparing the NPs to dilute THF solution. This suggests the preserved electronic structure in the aggregated state as well as the absence of the strong electronic coupling. More red-shifted absorption of TxNT compared to TxBT NPs is observed due to the stronger electron-accepting nature of the NT moiety compared to BT, as observed in THF.

### Fluorescence lifetimes

To obtain more detailed insights into the photophysical properties of the excited states, we examined time-resolved PL lifetimes of oligomers within NPs and in THF by using time-correlated single photon counting (TCSPC). In THF, the TxNT oligomer decayed monoexponentially with a lifetime *τ* = 3.3 ns. In contrast, TxBT in THF shows a biexponential decay with lifetimes *τ*_1_ = 0.64 ns (*A*_1_ = 0.8) and *τ*_2_ = 3.7 ns (*A*_2_ = 0.2) (Fig. S9).

Once in NPs, both oligomers demonstrate shortened monoexponential PL lifetimes compared to oligomers in THF with lifetimes of 1.2 ns for TxNT NPs and 0.72 ns for TxBT NPs (Fig. 2d). For TxBT, the monoexponential behaviour observed in NPs is in contrast to the biexponential decay in THF. This can be attributed to the short-lived component (*τ*_1_) being even shorter than the width of the instrument response function (IRF), as suggested by the fs-TA data below. This makes it difficult to resolve and leads to a best fit with a single exponential. The shortened PL lifetimes observed in NPs compared to the oligomers in THF suggests the presence of additional non-radiative deactivation pathways, likely arising from interchain and/or intersegment interactions within the NPs.

To investigate the role of the singlet excited state under conditions relevant for photocatalysis, we conducted fluorescence quenching experiments under conditions similar to those used for the HER experiments (Fig. S10). We performed steady-state and time-resolved PL quenching experiments by adding ascorbic acid (sacrificial donor, pH = 4.2) to the TxNT NPs and by *in situ* photodepositing Pt nanoparticles (co-catalyst) on the TxNT NP surfaces. No fluorescence lifetime quenching was observed upon the addition of either ascorbate or photodeposition of Pt NPs. Although the fluorescence lifetime remains unchanged after Pt photodeposition, a decrease in steady-state PL intensity is observed. This suggests static quenching or surface-related changes in the emissive properties of the TxNT NPs.

### Photocatalytic hydrogen evolution performance

To investigate the photocatalytic hydrogen evolution performance, we monitored the amount of hydrogen produced over 24 hours of continuous LED illumination (50 mW cm^−2^, 420–750 nm) for both TxNT and TxBT NPs. Ascorbic acid, which was predominately deprotonated at pH = 4.2, was used as a sacrificial electron donor, while Pt NPs photodeposited on the NP surfaces served as a co-catalyst electron acceptor (more details are provided in the SI).

**Fig. 3:**
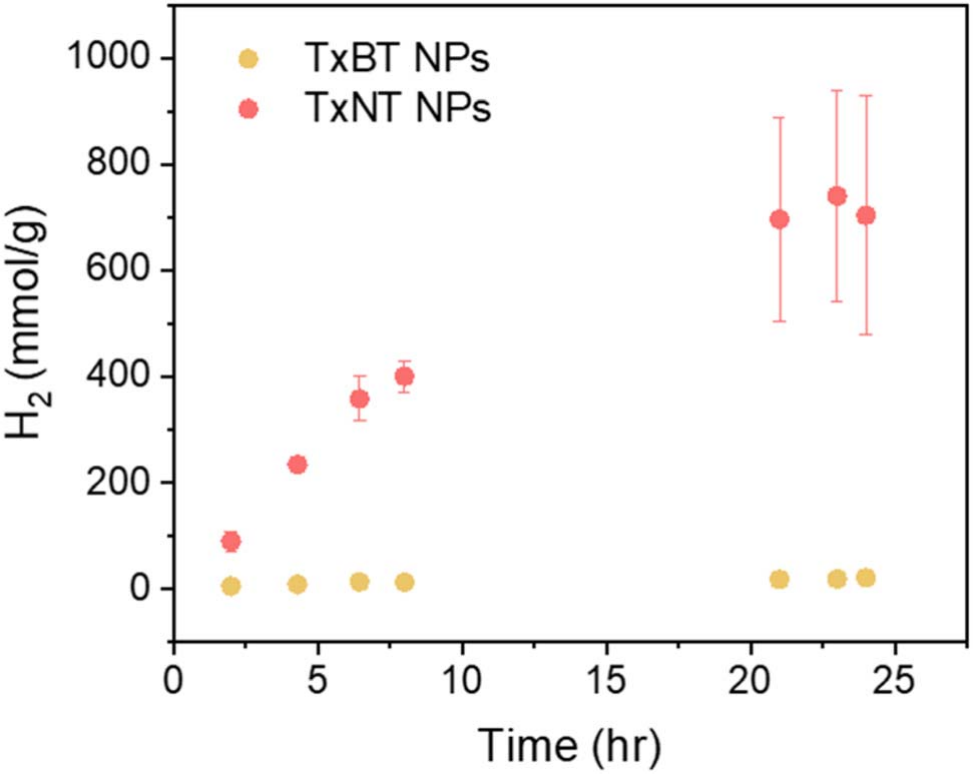
Photocatalytic hydrogen evolution performance of TxNT and TxBT NPs under continuous LED illumination for 24 hours in the presence of ascorbic acid (pH 4.2) as a sacrificial electron donor and photodeposited Pt as a co-catalyst.

The results show significant difference in HER activity between the two systems (Fig. 3). TxNT NPs showed a high initial HER rate of approximately 54 mmol h^−1^ g^−1^ and produced about 709 mmol g^−1^ of hydrogen after 24 hours of illumination. In contrast, TxBT NPs yielded only 18.5 mmol g^−1^ of hydrogen in 24 hours with an initial HER rate of 1.6 mmol h^−1^ g^−1^. TxNT NPs exhibited significantly higher HER activity, producing 35 times more hydrogen after 24 h than TxBT NPs. For both, TxNT and TxBT NPs the amount of hydrogen produced no longer increases after approximately 20 hours of illumination, indicating that photocatalyst stability remains a limiting factor. This behaviour may originate from inefficient charge hopping within the NPs to the surface, which can promote charge accumulation and subsequent photocatalyst degradation. Despite this limitation, the single-component TxNT NPs exhibit a HER rate of 54 mmol g^−1^ h^−1^, comparable to values reported for efficient heterojunction polymer NP photocatalysts (60–72 mmol g^−1^ h^−1^) under comparable conditions.^10,28,29^ Higher HER performance of TxNT NPs compared to TxBT NPs highlights the importance of molecular energetics and structure such as strong electron accepting nature and narrower bandgap.

### Transient absorption spectroscopy

**Fig. 4:**
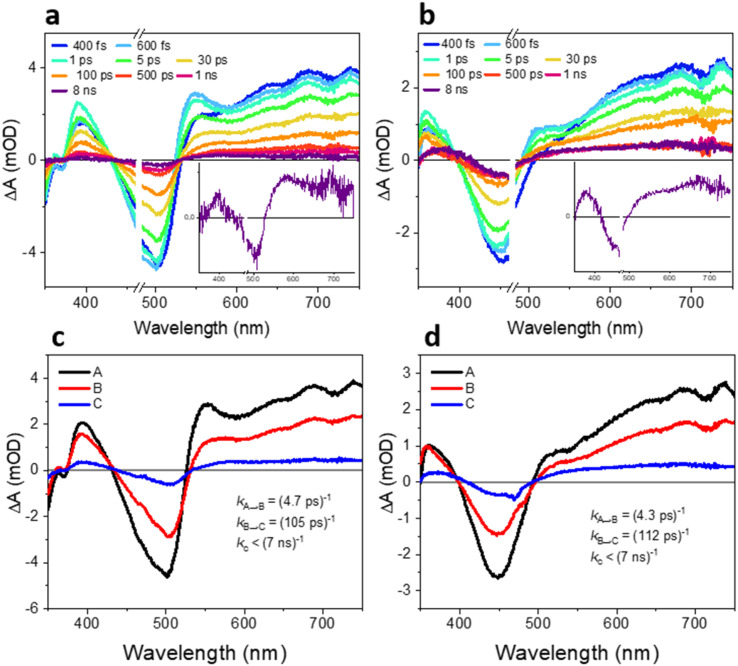
fs-TA spectra of (a) TxNT NPs and (b) TxBT NPs collected from 400 fs to 8 ns after 470 nm excitation (16.5 nJ per pulse) with insets showing the TA spectra recorded at 8 ns. EAS obtained from global analysis of the data presented in panels (a) and (b) for (c) TxNT and (d) TxBT NPs.

To further examine the reason behind the significantly better HER performance of TxNT compared to TxBT NPs, we performed femtosecond and nanosecond transient absorption (fs-TA and ns-TA) experiments to investigate the underlying excited-state and charge transfer dynamics. Fig. 4a and b shows fs-TA spectra probed from 400 fs to 8 ns after 470 nm excitation for both samples. Both TxNT and TxBT NPs show qualitatively similar features immediately after excitation: a ground-state bleach (GSB) at wavelengths corresponding to their steady-state absorption peaks, and excited-state absorption (ESA) bands in the blue and red parts of the spectra. TxNT NPs exhibit a GSB maximum around ∼500 nm, along with two ESA features: a peak at 400 nm and a broad band that emerges around 535 nm and extends into the red. TxBT NPs show a GSB around ∼440–450 nm, an ESA peak at ∼370 nm and an ESA band extending beyond ∼500 nm. Both NPs show contribution from stimulated emission on ps time scales, at the wavelengths of their steady-state PL maxima (a dip in the ESA around 560 and 580 nm, for TxBT and TxNT, respectively).

We further performed a global analysis of the fs-TA data, assuming two exponential steps and a long-lived photoproduct (states A → B → C) to obtain evolution-associated difference spectra (EAS) and time constants shown in Fig. 4c and d. The first relaxation process from state A to state B with a time constant of 4 ps could be assigned to vibrational cooling of the initially “hot” singlet excited state to thermally equilibrated (vibrationally relaxed) state for both TxNT and TxBT NPs, and a similar feature with *τ* ≈ 1.6 ps is present in the data in THF (Fig. S13 and S14). State B further decays within ∼105 and ∼112 ps for TxNT and TxBT NPs, respectively, forming long-lived species (state C) in both NPs that persisted beyond the 8 ns time window of our fs-TA setup. In both samples, state C is characterized by a further red-shift of the spectral features compared to states A and B, along with the disappearance of the ESA bands at 510 nm in TxBT NPs and 550 nm in TxNT NPs present in the early state spectra. Selected kinetic traces and concentration profiles from global analysis data of TxNT and TxBT NPs are shown in Fig. S11 and S12. Additional fs-TA spectra with the corresponding EAS for both oligomers in THF are presented in the SI (Fig. S13–S16). The observation of the long-lived state C motivated us to further investigate its nature in both samples, focusing on longer timescales ranging from tens of nanoseconds to microseconds.

**Fig. 5:**
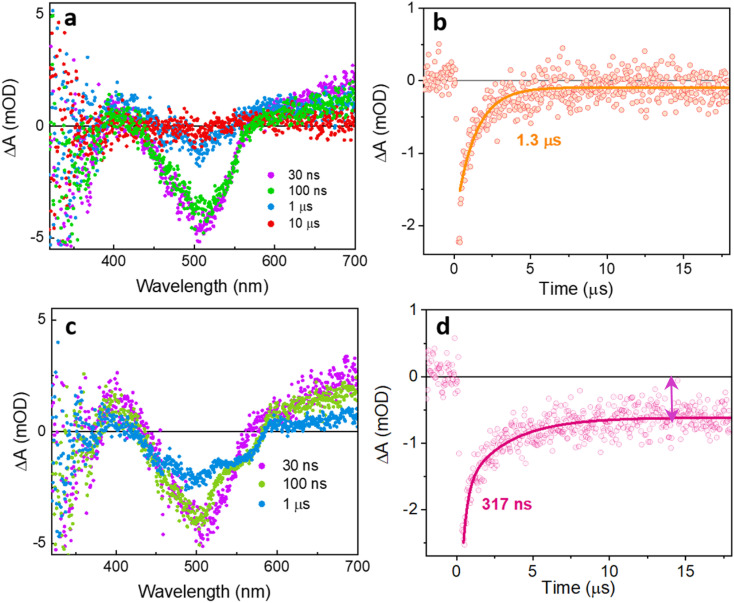
ns-TA spectra of pristine TxNT NPs (a) and TxNT NPs in the presence of ascorbic acid at pH 4.2 (c) at 470 nm excitation wavelength, 12 mJ per pulse. Kinetic traces at 510 nm show (b) monoexponential GSB recovery for pristine TxNT NPs (*τ* = 1.6 µs) and (d) biexponential recovery in the presence of ascorbic acid at pH 4.2 with a fast component (317 ns) and a long-lived offset indicated by the bidirectional arrow.

Using ns-TA spectroscopy, we probed the evolution of the long-lived state (state C) on timescales from tens of nanoseconds to hundreds of microseconds with 470 nm pulsed excitation. The spectral features of TxNT and TxBT NPs observed at 30 ns are consistent with the spectra obtained at 8 ns for both NPs (Fig. 5a, and S17a).

We first probed the dependence of the Δ*A* amplitude of the 750 nm ESA band for TxNT NPs (measured at 2.2 µs) on pump pulse energy (Fig. S18a). The amplitude of the 750 nm ESA feature scales linearly with the pump laser pulse energy (*R*^2^ ≈ 0.98). Additionally, the decay of the 750 nm ESA in TxNT NPs is monoexponential at all excitation densities used, with a lifetime in the range of ∼2–5 µs (Fig. S18b and c). The fitted lifetime showed some variations, especially at lower pulse energies due to the higher noise level in the data, but it shows no systematic power dependence. The GSB recovery at 510 nm for TxNT NPs was fit by a single exponential with *τ* ∼ 1.3 µs (Fig. 5b). The long lifetime, much longer than that of the fluorescent excited states of the NPs, suggests that it is either a local triplet state, or a charge separated state. Upon saturating the NP solution with oxygen, the lifetime of the signals at multiple wavelengths was reduced, in good agreement with triplet state quenching (Fig. S19). In contrast, electron transfer to oxygen from a charge separated state would have left signals from the oxidized donor, showing GSB. Additionally, direct detection of singlet oxygen phosphorescence revealed a singlet oxygen emission peak for TxNT NPs (Fig. S20).

We also recorded the transient spectra and the kinetics of TxBT NPs at several wavelengths under both oxygenated and deoxygenated conditions. Compared to TxNT NPs, TxBT NPs exhibited significantly more long-lived excited states (Fig. S17). Fitting the ns-TA TxBT NPs kinetics at multiple ESA wavelengths and GSB showed biexponential decay (Fig. S17b–d), one component on the order of few microseconds (∼1–5 µs) and another on the order of tens microseconds (roughly 30 µs). Saturating TxBT NP solution with oxygen also resulted in reduced lifetimes across multiple wavelengths, indicating the involvement of an oxygen-sensitive long-lived triplet (Fig. S21). Triplet energies were also estimated from DFT calculations for both oligomers as described in more detail in the SI.

To identify whether the triplets are localized or CT in character, we also performed relevant comparisons by obtaining ns-TA spectra and kinetics for both oligomers in solvents of different polarity (toluene and pyridine) at ambient temperature. Both oligomers showed significantly (*ca.* five-fold) longer triplet lifetimes in the more polar solvent pyridine compared to the nonpolar toluene (Fig. S22 and S23). As a purely localized triplet state would be less sensitive to solvent polarity, this could indicate a triplet CT character in both systems, as can be expected from their donor–acceptor structures. Additional oxygen quenching experiments for both TxNT and TxBT NPs showed a rapid decay of their transient signals, confirming efficient quenching of the excited triplet state (Fig. S24 and S25). We hypothesize that triplet CT could be relevant for the photocatalytic HER performance of TxNT and TxBT NPs.

Since we identified a long-lived state, assigned to a triplet CT state, in both oligomers in different solvents and NPs, we subsequently investigated how this state participates in the photocatalytic HER by performing quenching experiments under conditions similar to those for HER experiments. Two possible quenching pathways for the excited state were considered: (i) reductive quenching by the sacrificial donor (ascorbate), where the excited oligomer NP gets reduced by ascorbate; (ii) oxidative quenching by the Pt co-catalyst, where the excited oligomer NP transfers an electron to Pt and becomes oxidized.

To have a fair comparison, we kept the absorption at an excitation wavelength (470 nm) similar for all samples. We then compared the TA spectra and kinetics of pristine TxNT and TxBT NPs without the quencher present, in the presence of ascorbate with an adjusted pH to 4.2, with photodeposited Pt on the NP surfaces, and with both ascorbate (pH 4.2) and Pt present.

At 510 nm corresponding to GSB recovery, pristine TxNT NPs showed a 1.3 µs monoexponential recovery (Fig. 5b). In the presence of ascorbate at pH 4.2, distinct changes in the spectral shape were observed at 100 ns and 1 µs, suggesting the evolution of new transient species compared to pristine TxNT NPs (Fig. 5c). With the addition of ascorbate, the GSB recovery became biexponential with an initial fast recovery component of 317 ns followed by an offset signal that persisted beyond the 400 µs window (Fig. 5d and S26). A fast component is consistent with charge separation and subsequent charge recombination of the reduced TxNT and ascorbyl radical formed upon electron transfer to TxNT while the long-lived signal can be assigned to the reduced TxNT that accumulates and escapes recombination. Similarly, the kinetic trace at 750 nm in the presence of ascorbate showed a long-lived offset (Fig. S26). Introducing oxygen resulted in complete disappearance of the signal (Fig. S27). Additionally, the TA spectrum of TxNT NPs in the presence of ascorbate showed good agreement with spectroelectrochemical data obtained for the reduced TxNT oligomer in THF (Fig. S28). Photodeposition of the Pt co-catalyst on the surface of NPs did not result in any substantial changes in either spectrum or kinetics (Fig. S29). However, when both the Pt co-catalyst and ascorbate at pH 4.2 were present, the long-lived signal persisted, resembling the behaviour observed in the ascorbate-only case (Fig. S30). The Pt nanoparticles did not significantly quench the reduced TxNT intermediate on the timescale of our measurements. Based on these observations, the photocatalytic cycle for TxNT NPs can be described as follows: after 470 nm excitation, the TxNT NPs form a triplet-CT excited state within ∼100 ps; ascorbate in the solution then reductively quenches this state by donating an electron forming a reduced long-lived TxNT intermediate which eventually (*t* > 400 µs) transfers an electron to Pt on the NP surface, regenerating the ground state and allowing hydrogen evolution on the Pt.

Also, for TxBT NPs after ascorbate addition (Fig. S31), the kinetic trace at 490 nm corresponding to the GSB showed a long-lived offset. However, its amplitude was approximately half of that for TxNT NPs under photocatalytic conditions, and no significant changes were detected in the ESA kinetics at 600 nm (Fig. S31b), indicating less formation of long-lived intermediates in TxBT. Photodeposited Pt showed no observable quenching (Fig. S32). In the presence of both ascorbate and photodeposited Pt (pH 4.2), a long-lived component remained detectable but was reduced in amplitude compared to TxNT NPs under similar conditions (Fig. S33). This lower quenching efficiency likely arises from the smaller thermodynamic driving force for reduction by ascorbate at pH 4.2, since the E^*/−^ of TxBT is ∼0.3 V more positive than that of TxNT relative to NHE. These findings align with the results of the photocatalytic HER experiments, where only a small amount of hydrogen was detected for TxBT NPs. Overall, the results suggest that only a minor fraction of photoexcited states in TxBT NPs react productively, leading to a low overall efficiency.

We also performed cryo-TEM for both TxNT and TxBT NPs after Pt photodeposition, using a comparable amount of Pt precursor solution with respect to that in the HER experiments (Fig. S34). Cryo-TEM images revealed a pronounced Pt clustering on TxNT NPs, whereas TxBT NPs showed reduced Pt deposition on the surface. This observation, together with the reduced driving force for reduction by ascorbate, likely contributes to the significantly lower HER activity compared to TxNT NPs. The limited Pt loading reduces the number of active sites for proton reduction, leading to inefficient utilization of the photoexcited states and lower hydrogen production.

## Conclusions

We have developed two new star-shaped D–A oligomers, TxNT and TxBT, and demonstrated their structure-dependent performance in photocatalytic hydrogen evolution. TxNT NPs with a stronger electron-accepting NT unit, enabled significantly improved HER activity, achieving HER rates comparable to those of the most efficient heterojunction NP systems. Both systems formed long-lived triplet CT states and underwent reductive quenching by ascorbate; however, TxNT NPs formed a greater population of reduced species that accumulated and escaped recombination, as evidenced by the larger long-lived offset observed under photocatalytic conditions compared to TxBT NPs. Upon ascorbate addition, TxNT NPs showed a signal that persisted beyond hundreds of microseconds and matched the spectroelectrochemical signature of the reduced TxNT oligomer in THF. Cryo-TEM images further revealed a pronounced Pt photodeposition on TxNT NPs, while TxBT NPs showed significantly reduced Pt deposition. This suggests that a reduced number of active sites for proton reduction, combined with a smaller thermodynamic driving force for reduction by ascorbate of TxBT NPs, contributes to their significantly lower HER activity compared to TxNT NPs. These findings demonstrate that precise control of molecular energy levels, excited-state lifetimes, and NP morphology is critical for efficient solar hydrogen production. The results provide valuable design principles for next-generation organic photocatalysts exploiting long-lived triplet charge-transfer states.

## Author contributions

A. B. prepared NPs and performed DLS, steady-state absorption and PL, time-resolved PL, photocatalytic hydrogen evolution, fs- and ns-TA spectroscopy, carried out data analysis, curation, and interpretation, and wrote the original draft. G. K. synthesized the oligomers and performed NMR measurements. B. C. interpreted the NMR spectra, wrote the synthetic procedure, and performed singlet oxygen emission experiments. M. A. performed electrochemical and spectroelectrochemical measurements. M. V. P. conducted cryo-TEM measurements. L. K. designed, performed and interpreted the quantum chemical computations. L. H. contributed to discussion of the TA data. H. T. conceived the project and contributed to data analysis and interpretation. All authors reviewed and edited the manuscript.

## Conflicts of interest

There are no conflicts to declare.

## Supplementary Material

SC-OLF-D5SC09380E-s001

## Data Availability

The data supporting this article have been included as part of the supplementary information (SI). The raw data are available upon request from the authors. Supplementary information is available. See DOI: https://doi.org/10.1039/d5sc09380e.
