# Supplementary material for: Triplet states enable efficient photocatalytic hydrogen evolution in star-shaped truxene-based nanoparticles
Source: Chem Sci. 2026 Feb 5;17(15):7525–32. doi: 10.1039/d5sc09380e (PMC12919666; doi:10.1039/d5sc09380e)
Supplement: SC-017-D5SC09380E-s001 [file SC-017-D5SC09380E-s001.pdf]

Supplementary Information (SI) for Chemical Science.
This journal is © The Royal Society of Chemistry 2026

Supplementary Information for

Triplet States Enable Efficient Photocatalytic Hydrogen Evolution in Star-Shaped Truxene-Based Nanoparticles

Andjela Brnovic[1], Gaurav Kumar[1], Martin Axelsson[1], Bin Cai[1], Mariia V. Pavliuk[1], Lars Kloo[2], Leif Hammarström[1], Haining Tian[1*]

[1]Department of Chemistry - Ångström Laboratory, Uppsala University, Uppsala SE 75120, Sweden

[2]Applied Physical Chemistry, Department of Chemistry, KTH Royal Institute of Technology, SE-10044 Stockholm, Sweden

[*]Corresponding author: haining.tian@kemi.uu.se

Materials and Instrumentation

Truxene core ester benzeneboronic acid was synthesized according to our reported reference.[1] All chemicals were purchased from Sigma-Aldrich and used without purification unless specified. Proton nuclear magnetic resonance (^{1}H-NMR) and carbon-13 nuclear magnetic resonance (^{13}C NMR) were performed on Varian FT-NMR spectrometer (400 MHz). Polystyrene grafted with ethylene oxide and carboxyl groups (PS–PEG–COOH, backbone chain M_w 8500, graft chain M_w 4600, total chain M_w 36500) was purchased from Polymer Source. An Agilent 1260 Infinity Gel Permeation Chromatography (GPC) was used to measure the number-average molecular weights (M_n) and molecular weight distributions PDI (M_w/M_n) of the polymer samples. The GPC was fitted with PolyPore columns and a RI detector. The mobile phase was THF (1 mL min^{-1}) and the analysis was performed at 35 °C. PMMA standards were used to calibrate the system. Experiments and measurements were carried out at room temperature under standard ambient conditions.

Synthesis of TxBT and TxNT

697 mg Truxene core (0.5 mmol), 219 mg 4,7-dibromobenzo[1,2,5]thiadiazole (0.75 mmol) were dissolved in 50 mL DMF in a N_2 replaced three-neck bottle. Then, 1.3 mL 0.2 M Na_2CO_3 aqueous solution added. After purging the solution with N_2 for 30 mins, 70 mg $Pd(PPh_3)_4$ added into the mixture under N_2 purging. The mixture was heated at 110 °C overnight. After washing the mixture with water, and extracting with DCM, the organic solvent was removed via rotary evaporation. The crude product was purified with the silica chromatography, eluent: Heptane: EtOAc = 5:1. 510 mg red powder was obtained (yield 70%).

^{1}H NMR (400 MHz, $CDCl_3$) δ 8.59 – 8.34 (m, 1H), 8.22 – 7.96 (m, 1H), 7.88 – 7.62 (m, 1H), 7.41 (d, J = 46.8 Hz, 1H), 6.93 (dd, J = 38.7, 22.7 Hz, 1H), 3.85 – 3.40 (m, 2H), 3.19 – 2.69 (m, 2H), 1.51 – 1.12 (m, 20H), 0.99 – 0.60 (m, 10H).

^{13}C NMR (400 MHz, $CDCl_3$) δ 8.59 – 8.34 (m), 8.22 – 7.96 (m), 7.88 – 7.62 (m), 7.41 (d, J = 46.8 Hz), 6.93 (dd, J = 38.7, 22.7 Hz), 3.85 – 3.40 (m), 3.19 – 2.69 (m), 1.51 – 1.12 (m), 0.99 – 0.60 (m).

M_n = 8303 g/mol, PDI = 1.6

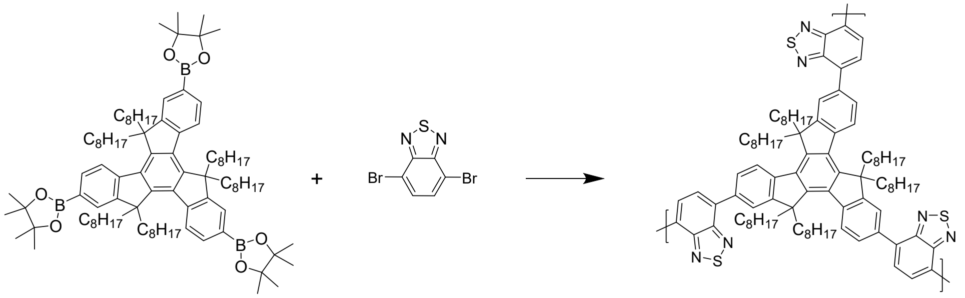

Figure S1. The synthetic route for TxBT.

697 mg Truxene core (0.5 mmol), 300 mg 5,10-dibromonaphtho[1,2-c:5,6-c']bis([1,2,5]thiadiazole) (0.75 mmol) were dissolved in 50 mL DMF in a N_2 replaced three-neck bottle. Then, 1.3 mL 0.2 M Na2CO3 aqueous solution was added. After purging the solution with N_2 for 30 min, 70 mg $Pd(PPh_3)_4$ was added into the mixture under N_2 purging. The mixture was heated at 110 ˚C overnight. After washing the mixture with water, and extracting with DCM, the organic solvent was removed via rotary evaporation. The crude product was purified with the silica chromatography, eluent: Heptane: EtOAc = 5:1. 446 mg red powder was obtained (yield 50%).

1H NMR (400 MHz, $CDCl_3$) δ 8.47 – 8.30 (m, 1H), 8.26 – 8.18 (m, 1H), 8.14 – 8.08 (m, 1H), 7.94 (ddd, J = 10.8, 7.4, 6.6 Hz, 1H), 7.78 (d, J = 7.7 Hz, 1H), 7.68 – 7.61 (m, 1H), 7.55 – 7.50 (m, 2H), 7.46 – 7.42 (m, 1H), 3.49 – 3.38 (m, 2H), 2.87 (t, J = 29.1 Hz, 2H), 1.36 – 1.27 (m, 20H), 0.86 (t, J = 6.8 Hz, 10H).

13C NMR (400 MHz, $CDCl_3$) δ 152.82 (s), 152.72 (s), 133.11 (s), 132.20 (s), 132.13 – 132.03 (m), 132.08 – 132.07 (m), 132.02 (s), 131.99 (s), 128.63 (s), 128.51 (s), 128.29 (s), 128.09 (s), 127.58 (s), 126.89 (s), 126.08 (s), 125.88 (s), 125.35 (s), 61.45 (s), 48.87 (s), 31.89 (s), 31.69 (s), 29.47 (s), 29.43 (s), 29.30 (s), 29.27 (s), 29.22 (s), 29.08 (s), 26.40 (s), 24.94 (s), 22.71 (s), 22.64 (s), 22.51 (s), 14.16 (s), 14.12 (s).

M_n = 9280 g/mol, PDI = 1.8

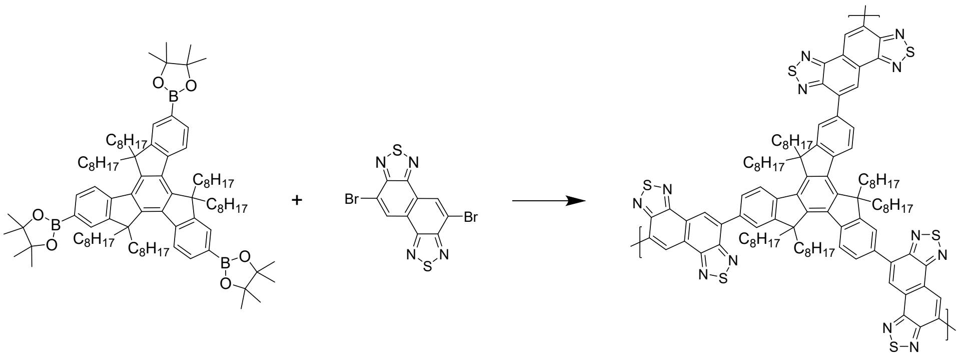

Figure S2. The synthetic route for TxNT.

Electrochemical measurements

All cyclic voltammetry measurements were performed in a cylindrical glass cell (⌀ 2.5 × 5 cm). The solvent used was THF (>99.7%, unstabilized, HPLC grade) kept dry over 3 Å molecular sieves, with 0.2 M recrystallized tetrabutylammonium hexafluorophosphate (TBA PF6) as the supporting electrolyte. As working electrode, a ⌀ 3 mm glassy carbon disk electrode was used, polished with a 0.05 μM Al particle paste in-between measurements. A Pt wire was used as a counter electrode. The reference electrode was a silver-wire (Ag/Ag^{+}) pseudo-reference in THF with the electrolyte in a glass tube, connected to the solution via a porous Vycore frit; the potential was then confirmed with ferrocene as an internal standard.

UV–vis spectroelectrochemistry (SEC) was performed using an Agilent 8453 diode array spectrophotometer in a 1 mm path-length quartz cuvette. As working electrode, a 1 × 1 × 0.1 cm platinum mesh was used with the same counter electrode and reference electrode as in the CV experiments. Controlled potential electrolysis was conducted using an Autolab PGSTAT100 potentiostat.

Preparation of TxBT and TxNT NPs

TxBT and TxNT NPs were prepared following nanoprecipitation procedure reported previously.[2] In brief, tetrahydrofuran (THF) stock solutions of the oligomer (0.1 mg mL^{-1}) and PS–PEG–COOH (1 mg mL^{-1}) were prepared and and mixed in a 1:3 wt/wt oligomer:PS–PEG–COOH ratio. A 4.5 mL oligomer:PS–PEG–COOH solution was rapidly injected into 12 mL of deionized water and sonicated for 5 min. THF was evaporated by heating the samples at 85 ˚C.

Dynamic Light Scattering (DLS) Measurements

Hydrodynamic diameters were measured using a Zetasizer Nano S from Malvern Instruments Nordic AB.

Cryogenic transmission electron microscopy (cryo-TEM)

Cryo-TEM samples were prepared following previously reported procedures.[3] Briefly, the TxNT and TxBT NPs, both with and without photodeposited Pt, were concentrated by centrifugation to a final concentration of 1000 µg mL^{-1}. The samples were then vitrified and imaged using a Zeiss Libra 120 transmission electron microscope (Carl Zeiss AG, Oberkochen, Germany) operated at 80 kV in zero-loss bright-field mode. Digital images were acquired under low-dose conditions using a BioVision Pro-SM slow-scan CCD camera (Proscan Elektronische Systeme GmbH, Scheuring, Germany).

Steady-State Spectroscopy

UV-Vis absorption spectra were measured using a Varian Cary 5000 UV-Vis-NIR spectrophotometer. Steady-state photoluminescence measurements were performed on a Fluorolog 3-222 spectrofluorometer (Horiba Jobin-Yvon) equipped with the FluorEssence software and using a 90° detection geometry. Emission data were corrected for variations in both the excitation source intensity and the detector sensitivity and the detector response.

Time-Correlated Single Photon Counting (TCSPC)

TCSPC measurements were performed using an FS5 spectrofluorometer (Edinburgh Instruments) equipped with a picosecond pulsed light-emitting diode (EPL-470) operating at an excitation wavelength of 470 nm. The instrument response function (IRF) was recorded using a diluted LUDOX® (Sigma-Aldrich) solution in deionized water, measured in a 1 cm path-length quartz cuvette at 470 nm emission to capture the scattered light profile. Sample absorbance was adjusted to 0.08 at 470 nm to minimize reabsorption and inner filter effects. Measurements were conducted at room temperature in THF or water. Data were analysed using DecayFit software.

Photocatalytic Hydrogen Generation

Photocatalytic hydrogen evolution experiments were conducted in airtight 9 mL vials. Each vial was loaded with 1.5 mL of a pre-diluted aqueous NPs solution (47 µg/mL), into which 10 µL of an aqueous potassium hexachloroplatinate (H_2PtCl_6) solution was added to achieve a 6 wt% Pt loading relative to the NP mass. The resulting mixtures were degassed by purging with argon (Ar) for 15 minutes. 0.5 mL of a 0.8 M L-ascorbic acid solution (pH 4.2, adjusted with 2 M KOH to ensure the presence of monodeprotonated ascorbate species) was introduced. The final reaction mixture was purged with Ar for an additional 30 minutes to eliminate residual dissolved oxygen. Samples were illuminated with 17 W LED PAR38 lamp (5000 K, Zenaro Lighting GmbH, $\lambda > 420$ nm). The irradiance at the sample surface was measured using a calibrated optical power and energy meter (Thorlabs PM100D), corresponding to 50 $mW\cdot cm^{-2}$. A cooling fan was used to dissipate excess heat and maintain a stable reaction temperature. The evolved hydrogen gas was quantified using a gas chromatograph (GC) system (TRACE 1300, Thermo Scientific) with Ar as the carrier gas. Gas sampling was performed from the headspace of the reaction vial at predefined time intervals. Each photocatalytic hydrogen evolution experiment was repeated three times under identical experimental conditions, and the reported values represent the average with standard deviations.

Transient Absorption (TA) Measurements

Femtosecond transient absorption (fs-TA) measurements were performed using a Ti:sapphire-based amplifier with an integrated oscillator and pump lasers (Coherent Libra). The 800 nm output (3 kHz, ~1.5 mJ/pulse) was split into a pump and probe by a beam splitter, which were directed toward the UV–vis–NIR TA spectrometer (TAS, Newport Corp.). The pump beam was passed through optical parametric amplifier (TOPAS-NIRUVis, Light Conversion) to obtain 470 nm wavelength. The pump beam was passed through a phase-locked chopper to reduce the repetition rate to 1.5 kHz, passed through depolarizer and attenuated with neutral density filter before reaching the sample. Probe supercontinuum was generated by focusing ~1 µJ of the fundamental onto calcium fluoride (CaF_2). The pump–probe delay was controlled via an optical delay line (temporal window $\leq$ 8 ns), and detection was performed using a silicon diode array (Newport Corp.). Samples were measured in quartz cuvettes (1 mm path length), and the optical density at the excitation wavelength was adjusted to ~0.1–0.6. Experiments were conducted in water or THF at room temperature. Pump powers were 50 and 100 µW

(equivalent to ~16.5–33 nJ/pulse), with the beam focused to a ~0.1 mm^2 spot on the sample. The instrument response function (IRF) was between 130 and 150 fs. To ensure data reliability, individual scans were examined for photodamage or inconsistencies.

All transient absorption (TA) data were processed using Surface Xplorer, which was used for background subtraction, time-zero correction, and for fitting the chirp to a third-order polynomial function. Kinetic analysis was performed using the R package TIMP and its GUI Glotaran. Global analysis was conducted by least-squares fitting of a sum of exponential components using a parallel kinetic model, where each component was associated with a distinct spectral feature. The resulting fits yielded evolution-associated spectra (EAS), representing the spectral evolution of excited-state species over time.

Nanosecond transient absorption (ns-TA) measurements were performed by using Nd:YAG laser/OPO combination (Ekspla, NT342B laser) that created ≈10 ns pulses at 1.8–18 mJ/pulse at 470 nm and 450 W ozone-free xenon arc lamp as the probe source. The probe beam was directed perpendicularly to the excitation laser as it passed through the sample. A symmetrical Czerny-Turner monochromator (TMS300) was used to select the detection wavelength. Kinetic traces at a single wavelength were recorded using an LP900 photomultiplier detector and subsequently digitized with a Tektronix TDS3012C oscilloscope. For full spectral acquisition, an Andor SH720 ICCD camera was employed. Data collection and processing were performed using the L900 software package. Measurements were performed at room temperature using a 1.0 cm path length quartz cuvette. Prior to measurements, all solutions were degassed with Ar.

DFT Computations

The molecules **TxBT** and **TxNT** were modelled using the hybrid density functional cam-B3LYP implemented in Gaussian 16 (Rev. C.01).[4,5] The molecular structure was geometrically optimized. Single-point, time-dependent calculations (TD-DFT) were made on the geometrically optimized structures using the same level of theory as for the geometry optimizations. 6-311G(d,p) basis sets were used for all the elements (H, C, N, O and S). Effects of implicit solvent effects were included using the dielectric properties of water (ε = 78.36) and toluene (ε = 2.37) in the polarizable continuum model (PCM).[6] Emission spectra were modelled based on all singlet-to-triplet transitions at lower energies than the first singlet-to-singlet absorption, with the assumption that the one lowest in energy is predominant in the radiative decay from the triplet state to the singlet ground state and that higher-energy transitions will contribute to a lower extent following a Boltzmann population relation.

Both monomeric molecular systems show complex structures, with three 'arms' of substituted entities extending from the aromatic molecular core (Figure S1). The molecules lack any global symmetry elements; thus, formally belonging to the symmetry group C_1. This is likely to result in rather complicated optical properties.

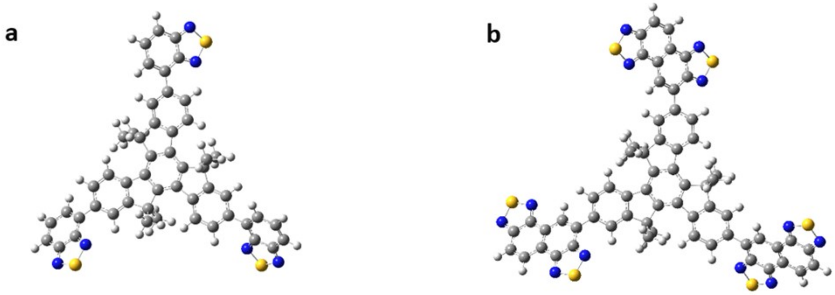

Figure S3. TxBT (a) and TxNT (b) geometrically optimized molecular structures, as obtained from computations not involving solvent effects.

Not including implicit solvent effects, **TxBT** displays a triplet ground state about 1.96 eV higher in energy than its singlet ground state. The molecule is therefore expected to be found in a singlet ground state. Since the electronic transition lowest in energy are expected to be dominated by contributions from the highest occupied molecular orbital (HOMO) to the lowest unoccupied molecular orbital (LUMO), this indicates that the lowest excitation energies should appear at about 2 eV. The HOMO energy of **TxBT** is -7.00 eV, and the LUMO energy is -1.43 eV. It is notable that the LUMO energies from DFT computations are notoriously unreliable. Results from TD-DFT computations show that the molecule experiences several very complex singlet→triplet transitions that are lowest in energy. These transitions involve several molecular orbitals (MOs) around the HOMO and LUMO (from HOMO-11 to LUMO+2). The lowest energy transition of the molecule is a singlet→triplet excitation ($S_0 \rightarrow T_1$) at 608.5 nm (2.04 eV, root 1). There are two close-by singlet→triplet excitatios at 601.3 nm and 600.3 nm, respectively. These can almost be regarded as a degenerate triple. All singlet→triplet transitions are formally spin-forbidden, and therefore the computations will provide oscillator strengths of zero. In complex molecules, such as **TxBT** and **TxNT**, the formal selection rules may partly be relaxed because of spin-orbit or vibronic coupling. However, if triplet states and/or phosphorescence are observed experimentally, a more likely scenario will involve an initial singlet→singlet excitation, followed by a radiation-less intersystem crossing to a long-lived triplet state with an ultimate radiative decay to the singlet ground state. The lowest energy singlet→singlet transition ($S_0 \rightarrow S_1$) is

observed at significantly higher energies, 344.8 nm (3.60 eV, $f = 0.77$, root 10) and is just like the $S_0 \rightarrow T_1$ transition involves a multitude of contributions from HOMO-4 to LUMO+2. Another near-degenerate transition can be found at 343.8 nm (3.61 nm, $f = 0.75$, root 11). Yet another and similar double transition is found at 280.0 nm (4.43 eV, $f = 0.35$, root 22) and 279.9 nm (4.43 eV, $f = 0.29$, root 23). In conclusion, excitation of **TxBT** at about 350 nm could cause an emission at just over 600 nm. The implicit solvent effects of toluene are expected to be marginal because of its low dielectric constant. The $S_0 \rightarrow T_1$ transition is observed at 604.9 nm and the $S_0 \rightarrow S_1$ at 347.4 nm and the higher energy singlet→singlet excitation at 282.3 nm. The corresponding values obtained including the dielectric effects of water are 599.1 nm, 348.8 nm and 285.7 nm, respectively. The only notable difference observed when including the dielectric fields of the solvents is a slightly higher oscillator strength for the $S_0 \rightarrow S_1$ transition in water.

The HOMO and LUMO energies of **TxNT** are -7.14 eV and -3.53 eV, respectively, and the difference between a singlet and triplet ground state 1.84 eV. The lowest energy ($S_0 \rightarrow T_1$) is found at 674.7 nm (1.84 eV, root 1) with nearby singlet→triplet excitations at 670.0 nm and 669.3 nm. The lowest energy singlet→singlet transition is observed at 383.9 nm (3.23 eV, $f = 0.80$, root 13) with a closely placed transition at 383.7 eV (3.23 eV, $f = 0.98$, root 14). At slightly higher energies the following pair of singlet→singlet transitions are found: 303.0 nm (4.09 eV, $f = 0.22$, root 22) and 302.4 nm (4.10 eV, $f = 0.19$, root 23). Including implicit solvent effects, the $S_0 \rightarrow T_1$ transition is observed at 668.6 nm in toluene and 660.1 nm in water, the $S_0 \rightarrow S_1$ at 386.5 nm in toluene and 387.1 nm in water, and the higher energy singlet→singlet excitation at 307.3 nm in toluene and 315.2 nm in water.

In the computed absorption and emission for **TxNT** and **TxBT**, the absorption and emission follow the main features of the experimentally obtained spectra (Figure 2c) shown below (no implicit solvent effects included) (Figure S4). Formally, and normally, the experimentally obtained emission is expected to arise because of vibrationally relaxed $S_1 \rightarrow S_0$ (Stokes-shifted) decay; *i.e.* because of fluorescence. For the currently studied molecules this may of course also be the case, although a fast non-radiative transition followed by a rather fast $T_1 \rightarrow S_0$ decay could provide similar results.

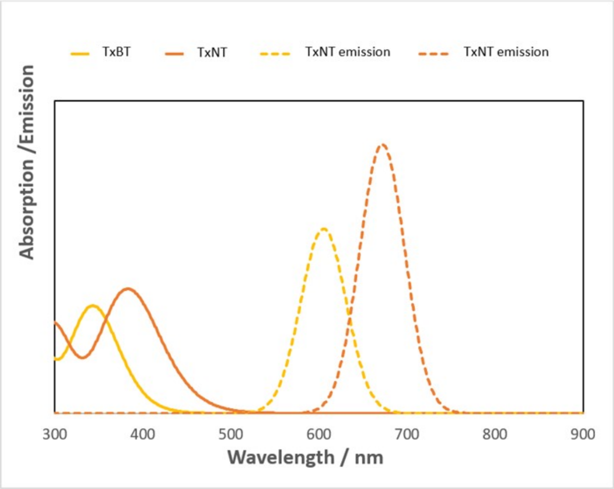

Figure S4. The computed UV-vis absorption and emission spectra of TxNT and TxBT without solvent effects included.

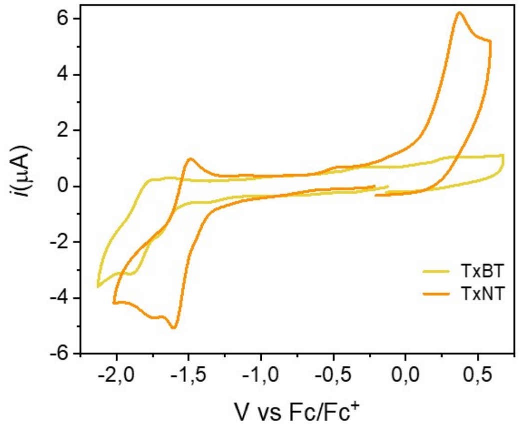

Figure S5. CV measurements of TxNT and TxBT oligomers in THF against the Fc/Fc^{+} redox couple.

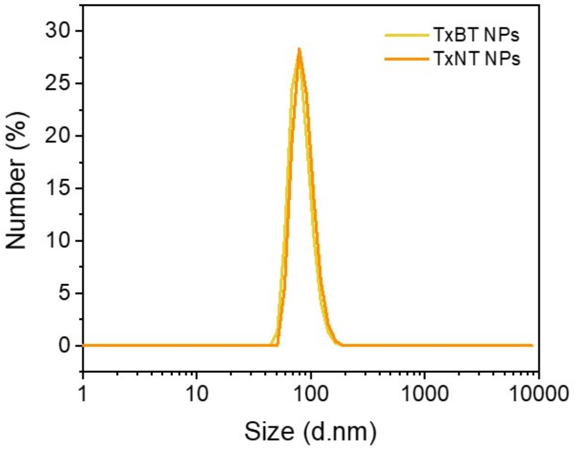

Figure S6. DLS measurements of TxBT and TxNT NPs in water. The hydrodynamic diameters were determined to be approximately 90 nm for TxBT NPs and 80 nm for TxNT NPs.

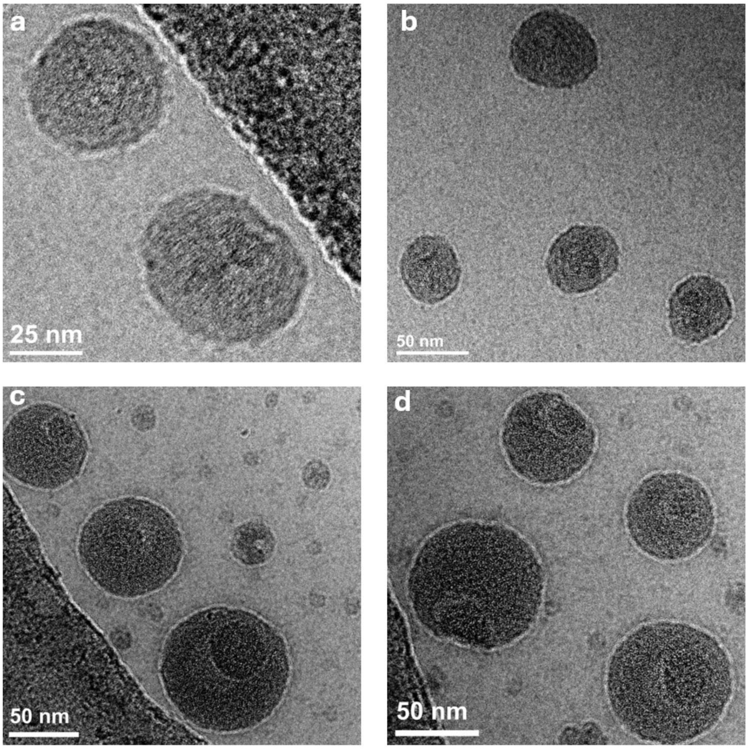

Figure S7. Cryo-TEM images of TxNT NPs (a, b) and TxBT NPs (c, d).

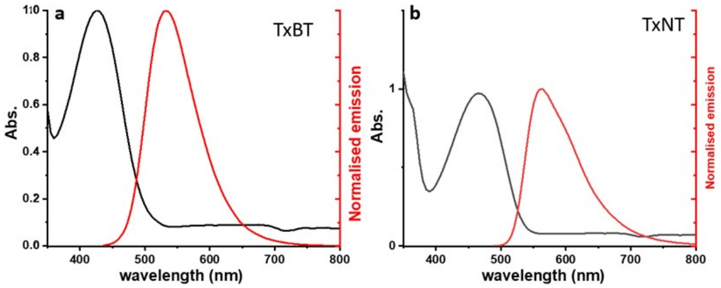

Figure S8. UV-Vis absorption and PL spectra of (a) TxBT and (b) TxNT in THF.

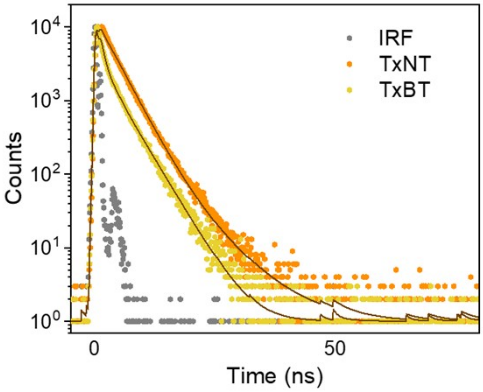

Figure S9. TCSPC decay curves of TxNT and TxBT oligomers in THF, along with corresponding fits convoluted with IRF.

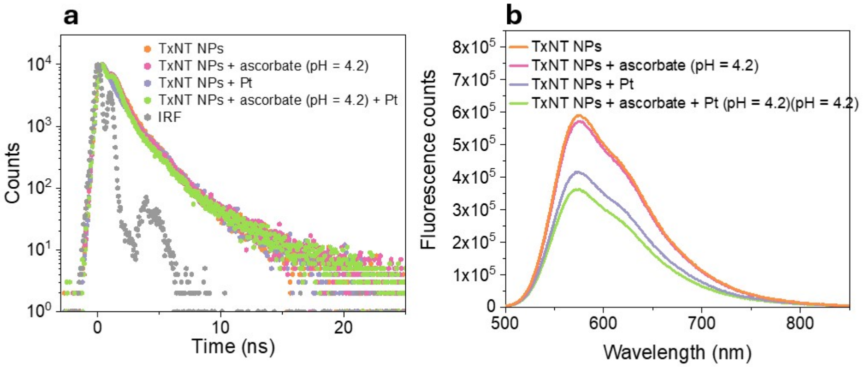

Figure S10. Time-resolved (a) and steady-state (b) PL quenching experiments on TxNT NPs with ascorbic acid (pH 4.2) and in situ photodeposited Pt nanoparticles.

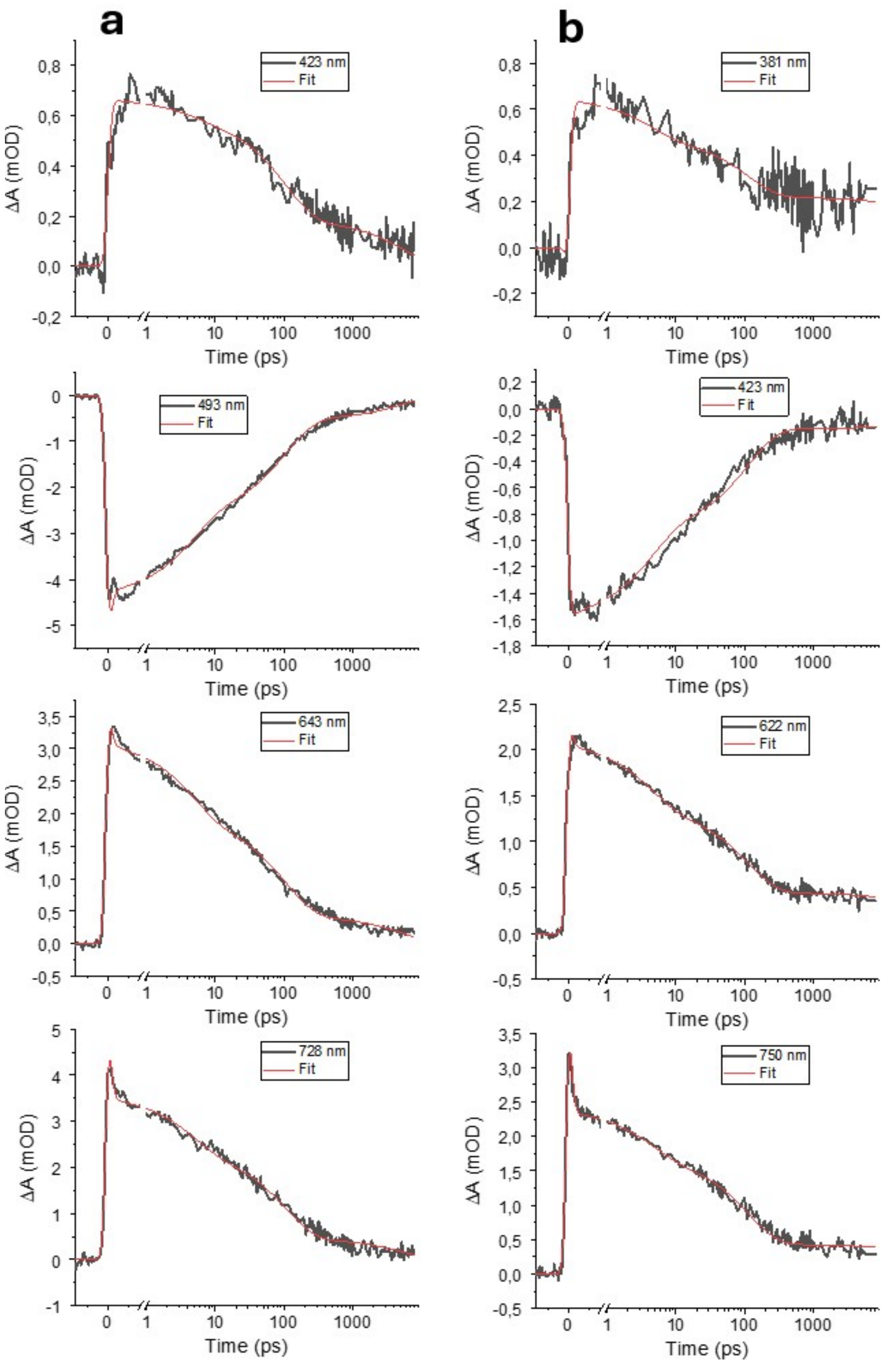

Figure S11. Selected kinetic traces from the global analysis in Figure 4c for TxNT NPs (a) and Figure 4d for TxBT NPs (b).

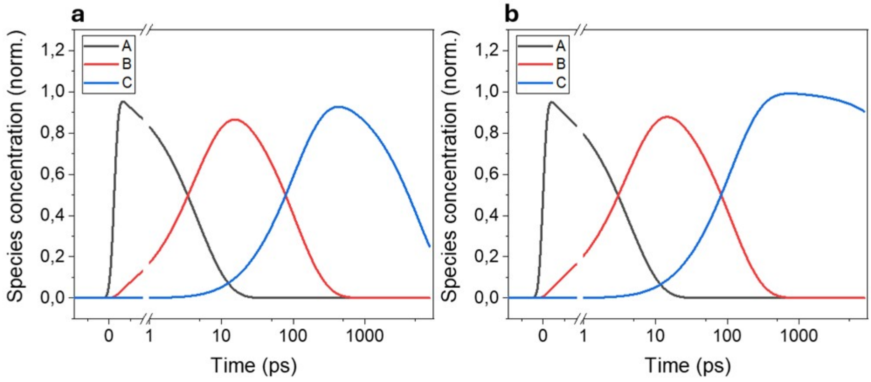

Figure S12. Species concentration profiles obtained from global analysis of fs-TA data for (a) TxNT NPs and (b) TxBT NPs. The data were fitted using a sequential kinetic model (A → B → C). The plots show the normalized population dynamics of species A (black), B (red), and C (blue) over time.

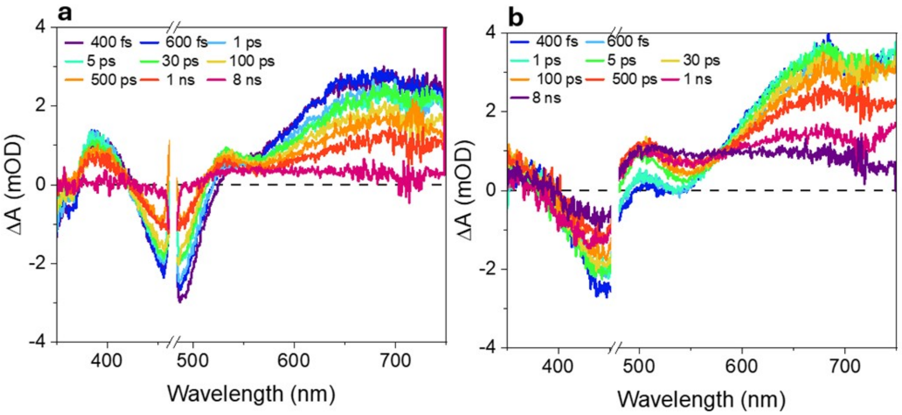

Figure S13. fs-TA spectra of TxNT (a) and TxBT (b) oligomers in THF upon 470 nm excitation (36.3 nJ/pulse), showing the evolution of spectral features over time from 400 fs to 8 ns.

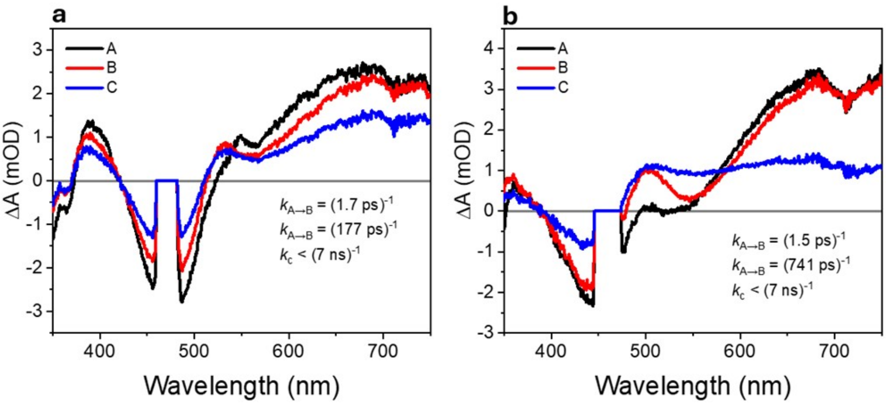

Figure S14. EAS obtained from global analysis of the data presented in Figure S13 for (c) TxNT and (d) TxBT oligomers in THF.

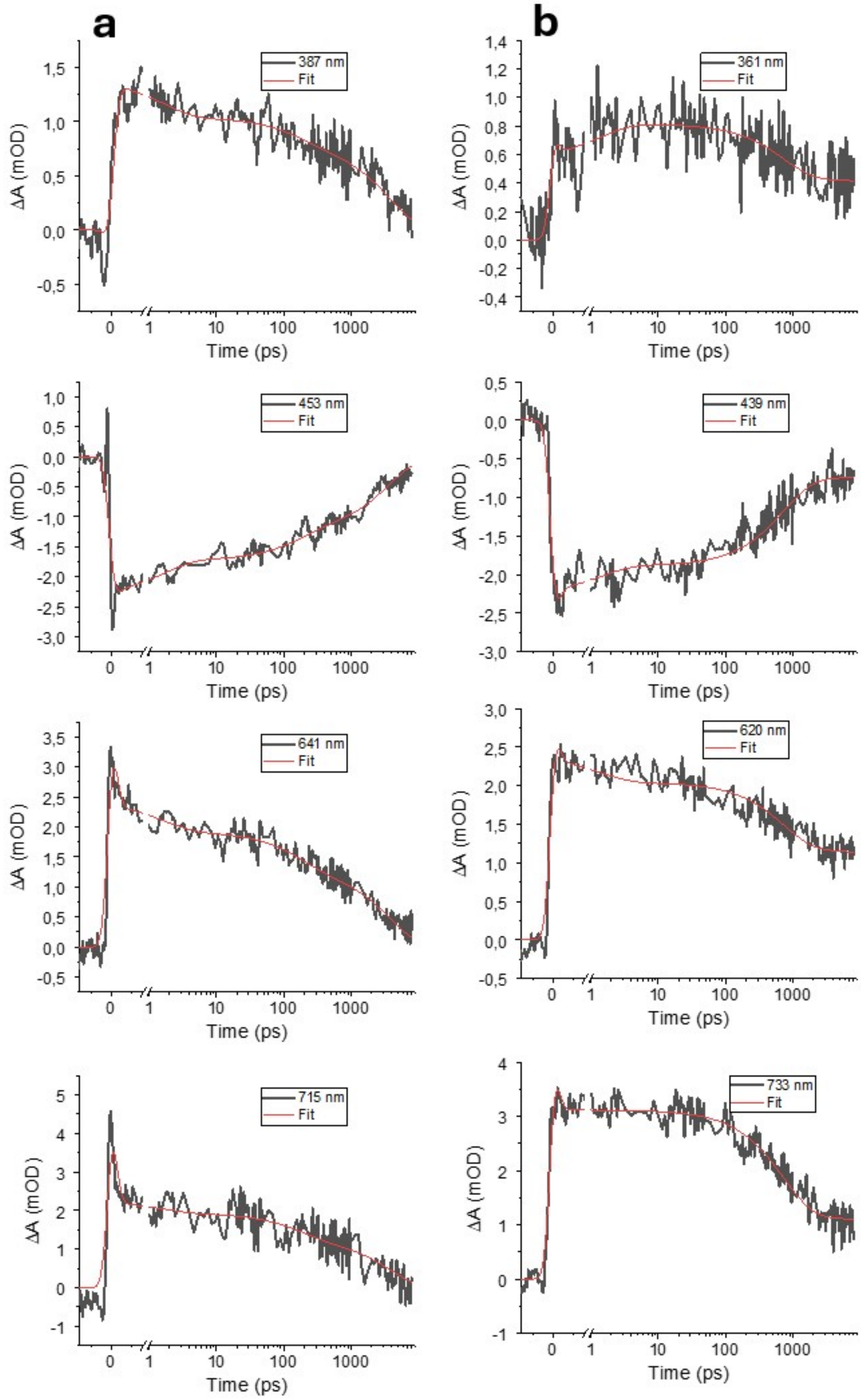

Figure S15. Selected kinetic traces from the global analysis in Fig. S14a for TxNT (a) and Figure S13b for TxBT (b) oligomers in THF.

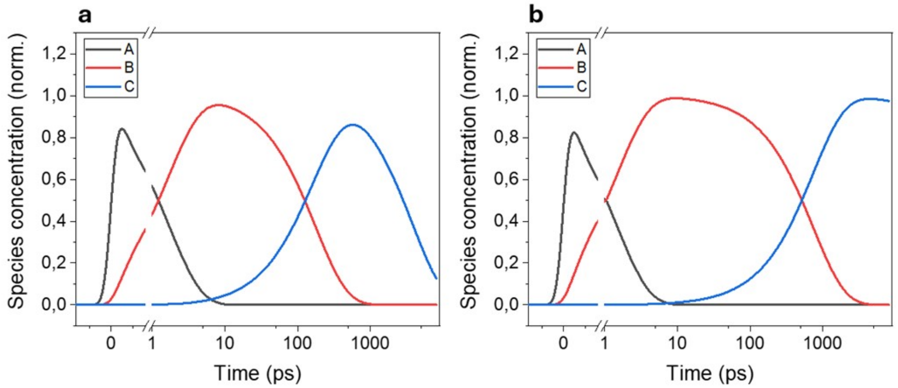

Figure S16. Species concentration profiles obtained from global analysis of fs-TA data for (a) TxNT and (b) TxBT oligomers in THF. The data were fitted using a sequential kinetic model (A → B → C). The plots show the normalized population dynamics of species A (black), B (red), and C (blue) over time.

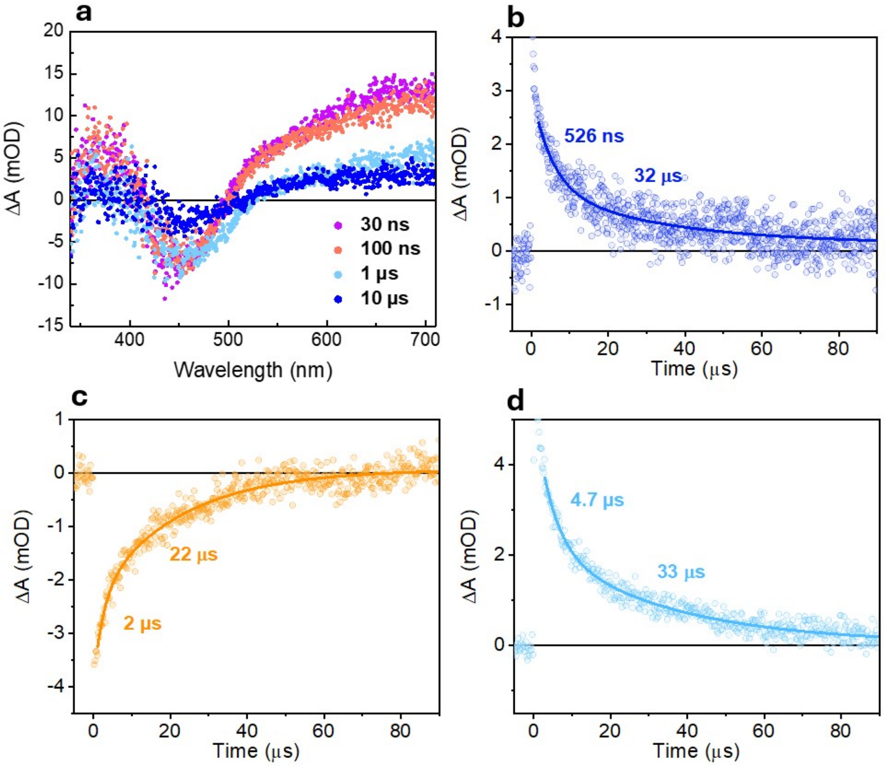

Figure S17. ns-TA spectra of TxBT NPs following 470 nm excitation (12 mJ/pulse), (a) Spectral evolution from 30 ns to 10 µs. (b–d) Kinetic traces extracted at 370 nm (b), 490 nm (c), and 600 nm (d), fit with biexponential functions.

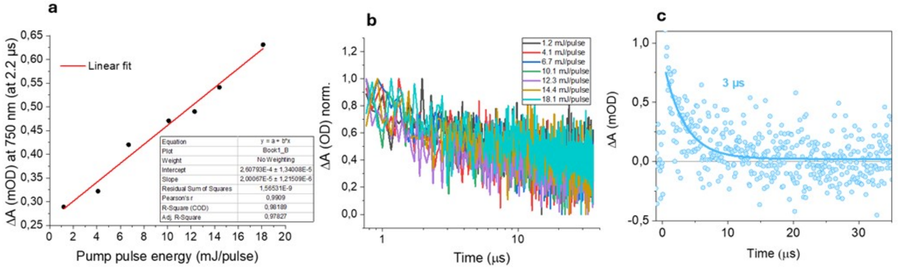

Figure S18. (a) Plot of ΔA amplitude at 750 nm (measured at 2.2 µs) versus pump pulse energy for TxNT NPs. A linear dependence is observed with $R^2 = 0.98$. (b) Normalized decay traces of the 750 nm ESA signal at different excitation pulse energies. The decay is monoexponential across all pulse energies, with lifetimes ranging from ~2 to 5 µs. Variations in fitted lifetimes at lower pulse energies are attributed to increased noise. (c) Representative decay trace (12 mJ/pulse) fitted with a monoexponential function, yielding a lifetime of ~3 µs.

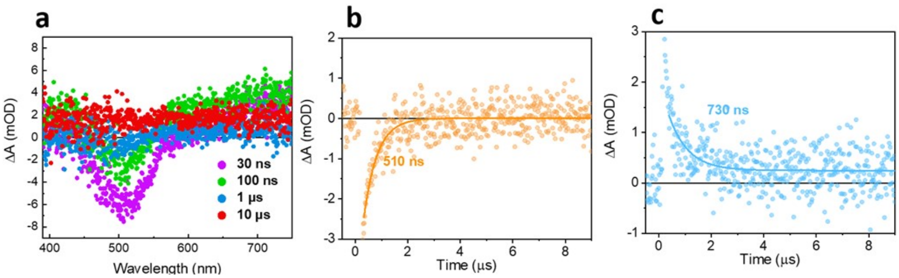

Figure S19. (a) ns-TA spectra of TxNT NPs recorded at selected time delays (30 ns to 10 µs) following 470 nm excitation under oxygen-saturated conditions. (b) Kinetic trace at 510 nm fitted with a monoexponential function, showing a reduction in lifetime (~510 ns). (c) Kinetic trace at 750 nm, also showing lifetime reduction (~730 ns) (12 mj/pulse).

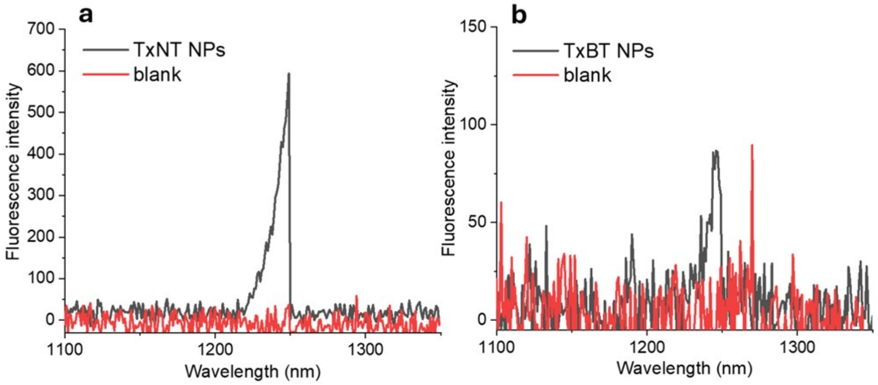

Figure S20. Singlet oxygen emission for TxNT (a) and TxBT (b) NPs in water upon 520 nm excitation

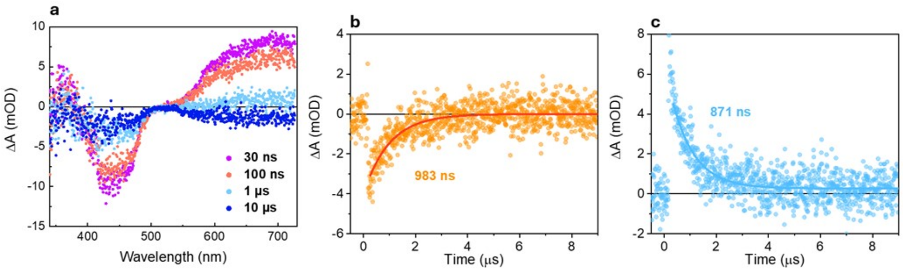

Figure S21. (a) ns-TA spectra of TxBT NPs recorded at selected time delays (30 ns to 10 μs) following 470 nm excitation under oxygen-saturated conditions. (b) Kinetic trace at 490 nm fitted with a monoexponential function, showing a reduction in lifetime (~983 ns). (c) Kinetic trace at 600 nm, also showing lifetime reduction (~871 ns) (12 mJ/pulse).

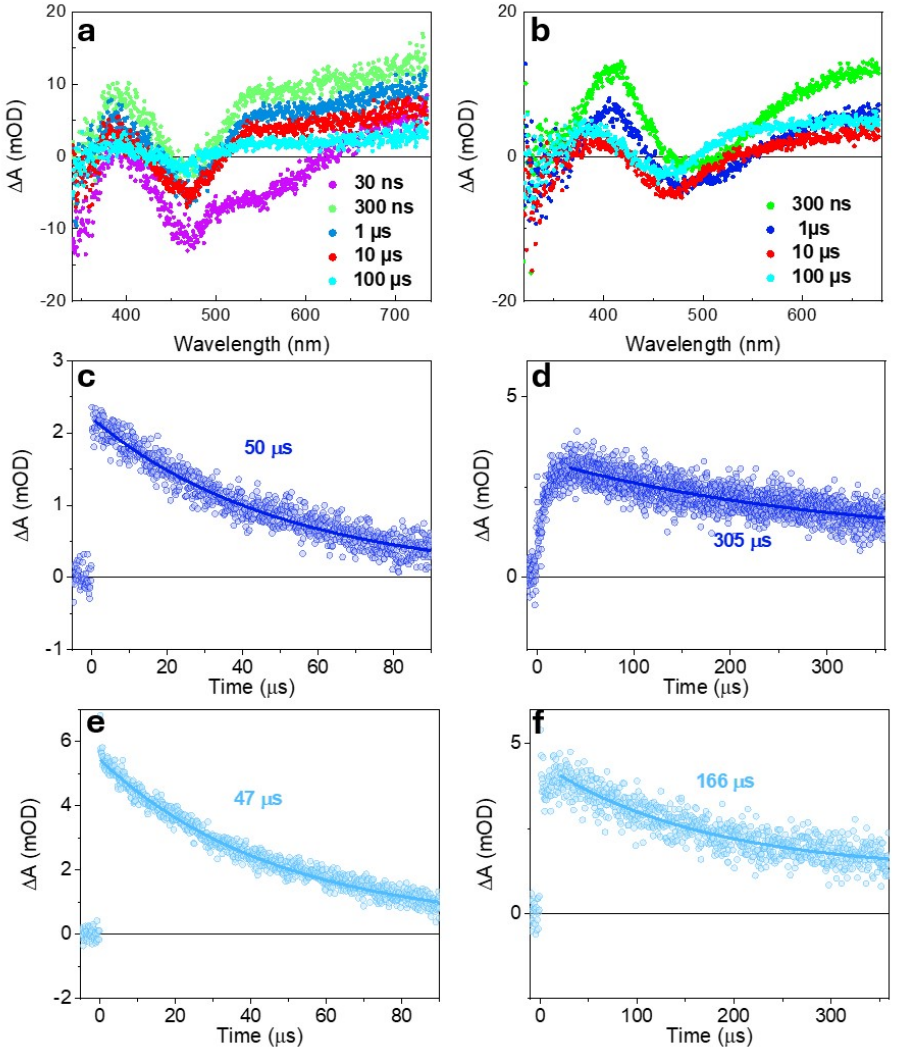

Figure S22. ns-TA spectra and kinetic traces of TxNT dissolved in toluene (left panels) and pyridine (right panels), excited at 470 nm (12 mJ/pulse). (a, b) Spectral evolution at selected time delays from 30 ns to 100 μs. (c, d) Kinetic traces at 380 nm in toluene and pyridine, respectively. (e, f) Kinetic traces at 700 nm in toluene and pyridine, respectively.

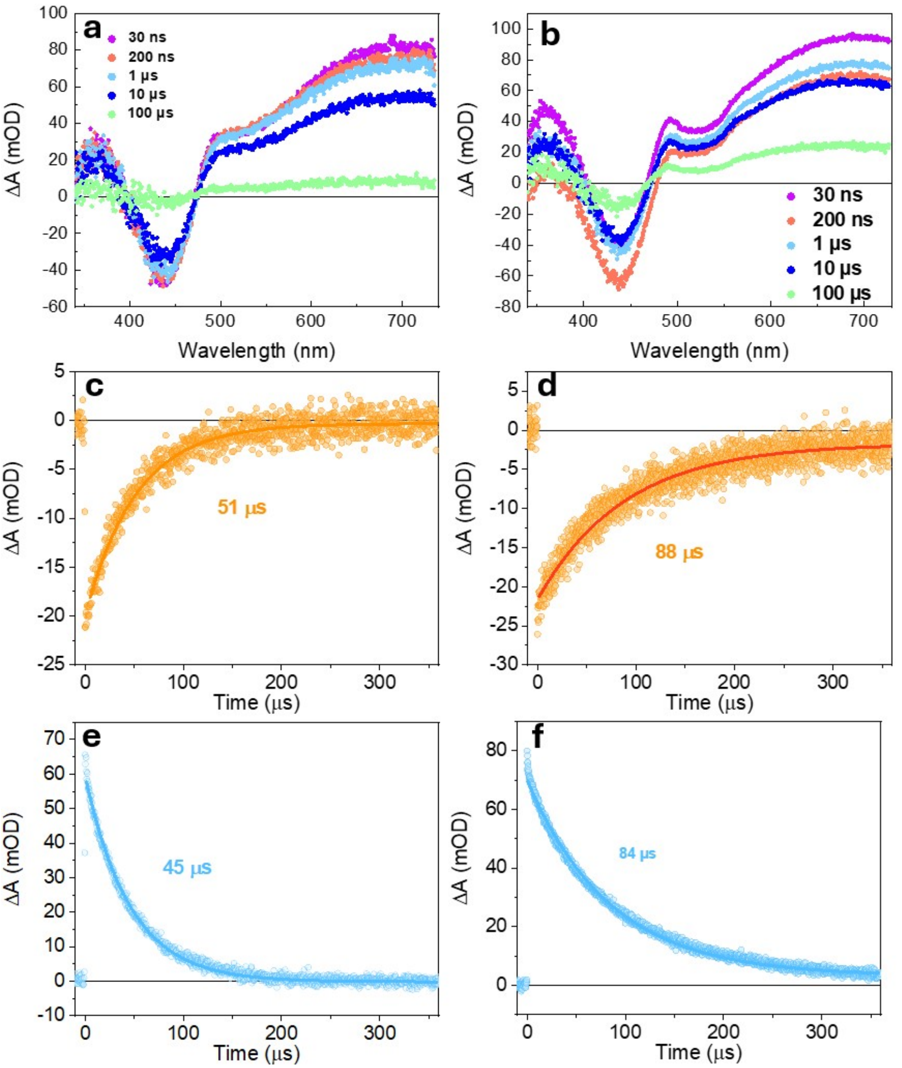

Figure S23. ns-TA spectra and kinetic traces of TxBT dissolved in toluene (left panels) and pyridine (right panels), excited at 470 nm (12 mJ/pulse). (a, b) Spectral evolution at selected time delays from 30 ns to 100 µs. (c, d) Kinetic traces at 420 nm in toluene and pyridine, respectively. (e, f) Kinetic traces at 700 nm in toluene and pyridine, respectively.

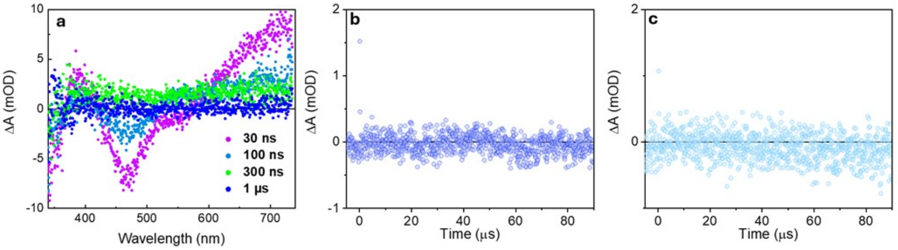

Figure S24. ns-TA data for TxNT oligomer in toluene saturated with oxygen (12 mJ/pulse). (a) Spectral evolution from 30 ns to 1 µs following 470 nm excitation. (b–c) Kinetic traces at 380 nm (b) and 700 nm (c).

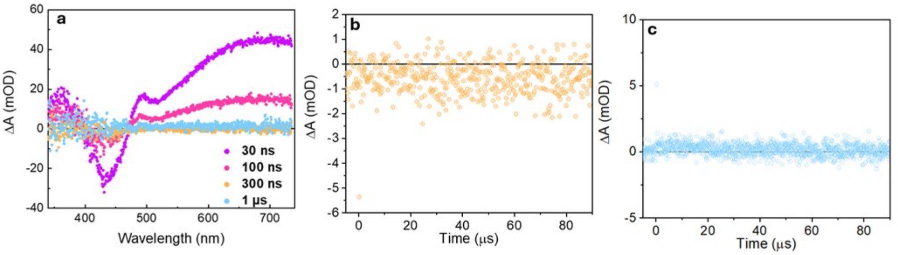

Figure S25. ns-TA data for TxBT oligomer in toluene saturated with oxygen (12 mJ/pulse). (a) Spectral evolution from 30 ns to 1 µs following 470 nm excitation. (b–c) Kinetic traces at 420 nm (b) and 700 nm (c).

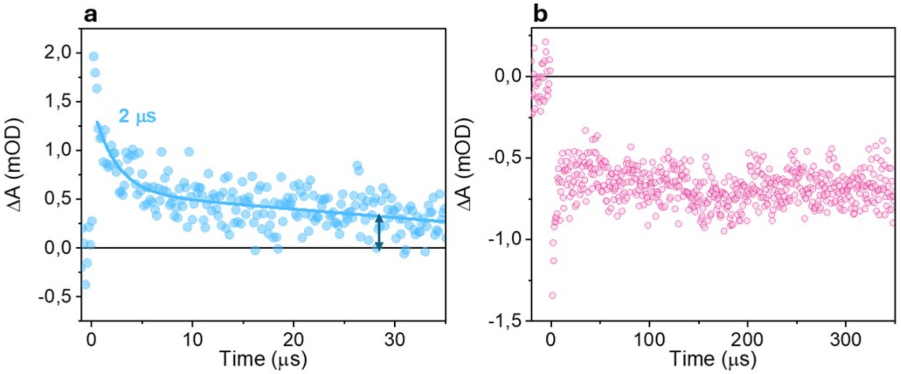

Figure S26. Kinetic traces of TxNT NPs in the presence of ascorbate, measured following 470 nm excitation (12 mJ/pulse). (a) A decay (750 nm) with a lifetime of ~2 µs is observed, followed by a long-lived offset extending beyond the measurement window. (b) GSB at 410 nm with incomplete recovery within the time scale of the measurement (~300 µs).

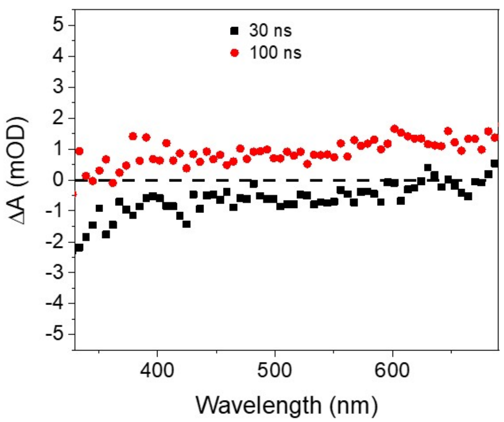

Figure S27. ns-TA spectra of TxNT NPs in the presence of ascorbate, measured at 30 ns and 100 ns after 470 nm excitation (12 mJ/pulse) shows completely quenched transient signal upon saturating with oxygen.

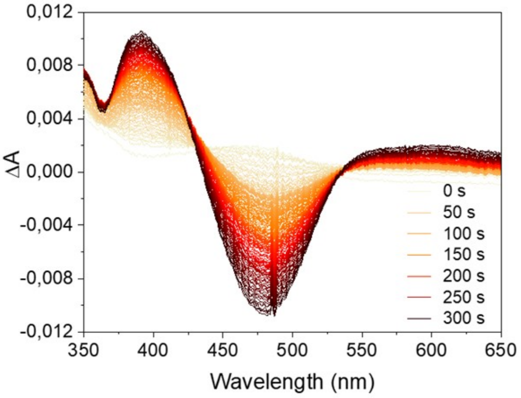

Figure S28. SEC absorption spectra of TxNT oligomer in THF during electrochemical reduction over time.

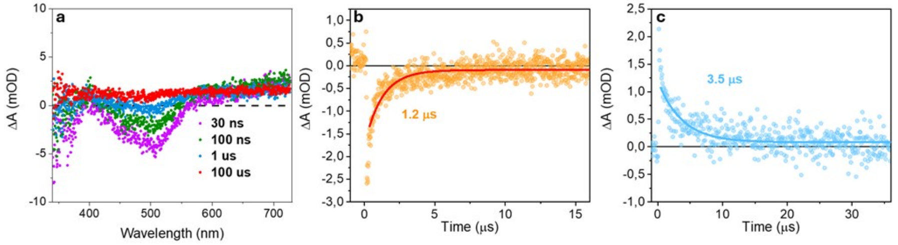

Figure S29. ns-TA data of TxNT NPs after Pt photodeposition (470 nm excitation, 12 mJ/pulse): (a) spectral evolution from 30 ns to 100 µs; (b) kinetics at 510 nm, and (c) kinetics at 750 nm.

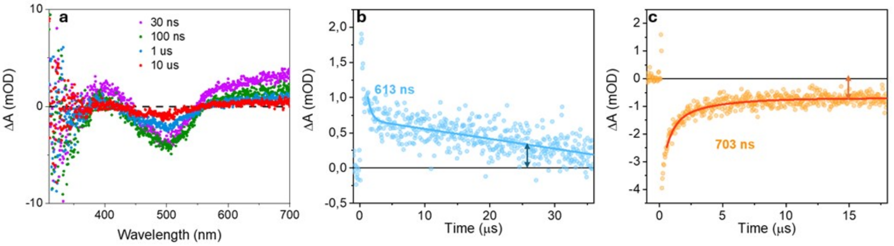

Figure S30. ns-TA data of TxNT NPs after Pt photodeposition and addition of ascorbate at pH 4.2 (470 nm excitation, 12 mJ/pulse): (a) spectral evolution from 30 ns to 100 µs; (b) kinetics at 510 nm, and (c) kinetics at 750 nm.

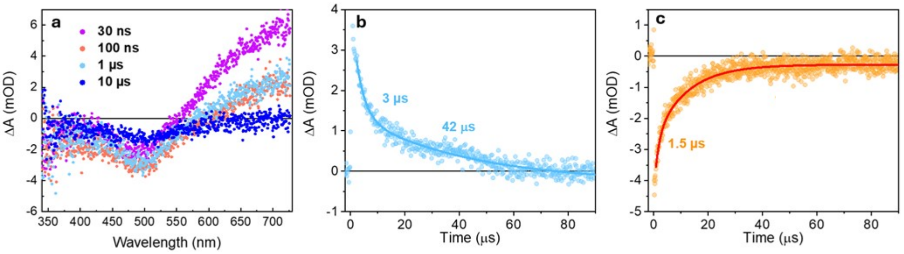

Figure S31. ns-TA data of TxBT NPs after addition of ascorbate at pH 4.2 (470 nm excitation, 12 mJ/pulse): (a) spectral evolution from 30 ns to 100 µs; (b) kinetics at 600 nm, and (c) kinetics at 490 nm.

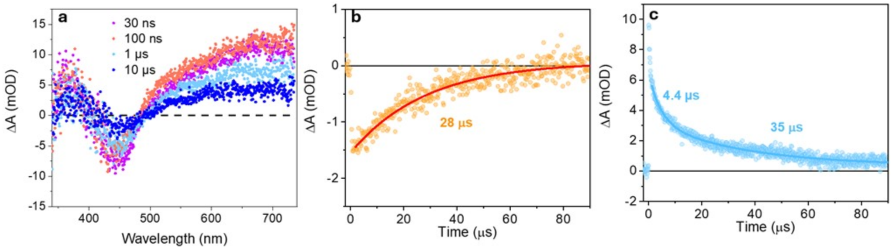

Figure S32. ns-TA data of TxBT NPs after Pt photodeposition (470 nm excitation, 12 mJ/pulse): (a) spectral evolution from 30 ns to 100 µs; (b) kinetics at 490 nm, and (c) kinetics at 600 nm.

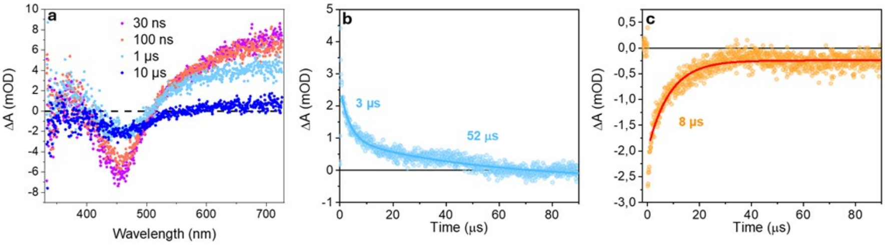

Figure S33. ns-TA data of TxBT NPs after Pt photodeposition and addition of ascorbate at pH 4.2 (470 nm excitation, 12 mJ/pulse): (a) spectral evolution from 30 ns to 100 µs; (b) kinetics at 490 nm, and (c) kinetics at 600 nm.

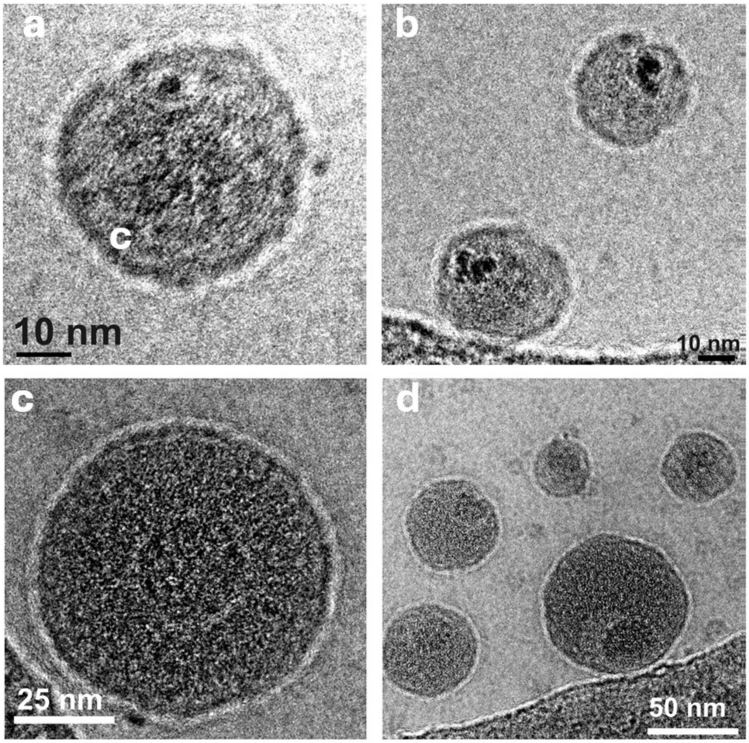

Figure S34. Cryo-TEM images of TxNT NPs (a, b) and TxBT NPs (c, d) after Pt photodeposition.

References

1 S. Wang, G. Kumar and H. Tian, *Solar RRL*, 2022, **6**, 2200755.

2 L. Wang, R. Fernández-Terán, L. Zhang, D. L. A. Fernandes, L. Tian, H. Chen and H. Tian, *Angewandte Chemie International Edition*, 2016, **55**, 12306–12310.

3 M. Almgren, K. Edwards and G. Karlsson, *Colloids Surf A Physicochem Eng Asp*, 2000, **174**, 3–21.

4. M. J. Frisch, G. W. Trucks, H. B. Schlegel, G. E. Scuseria, M. A. Robb, J. R. Cheeseman, G. Scalmani, V. Barone, G. A. Petersson, H. Nakatsuji, X. Li, M. Caricato, A. V. Marenich, J. Bloino, B. G. Janesko, R. Gomperts, B. Mennucci, H. P. Hratchian, J. V. Ortiz, A. F. Izmaylov, J. L. Sonnenberg, D. Williams-Young, F. Ding, F. Lipparini, F. Egidi, J. Goings, B. Peng, A. Petrone, T. Henderson, D. Ranasinghe, V. G. Zakrzewski, J. Gao, N. Rega, G. Zheng, W. Liang, M. Hada, M. Ehara, K. Toyota, R. Fukuda, J. Hasegawa, M. Ishida, T. Nakajima, Y. Honda, O. Kitao, H. Nakai, T. Vreven, K. Throssell, J. A. Montgomery, Jr., J. E. Peralta, F. Ogliaro, M. J. Bearpark, J. J. Heyd, E. N. Brothers, K. N. Kudin, V. N. Staroverov, T. A. Keith, R. Kobayashi, J. Normand, K. Raghavachari, A. P. Rendell, J. C. Burant, S. S. Iyengar, J. Tomasi, M. Cossi, J. M. Millam, M. Klene, C. Adamo, R. Cammi, J. W. Ochterski, R. L. Martin, K. Morokuma, O. Farkas, J. B. Foresman, and D. J. Fox, Gaussian16 (Rev. B.01), Gaussian, Inc., Wallingford CT, 2016.

5. T. Yanai, D. Tew, N. Handy, *Chem. Phys. Lett.*, 2004, **393,** 51-57.

6. J. Tomasi, B. Mennuci, R. Cammi, *Chem. Rev.*, 2005, **105,** 2999–3093.
